# *Corynebacterium pseudotuberculosis*: Whole genome sequencing reveals unforeseen and relevant genetic diversity in this pathogen

**DOI:** 10.1371/journal.pone.0309282

**Published:** 2024-08-26

**Authors:** Ekkehard Hiller, Verena Hörz, Reinhard Sting

**Affiliations:** 1 Chemical and Veterinary Analysis Agency Stuttgart, Fellbach, Germany; 2 Consiliary Laboratory for *Corynebacterium Pseudotuberculosis*, Fellbach, Germany; Kafrelsheikh University Faculty of Veterinary Medicine, EGYPT

## Abstract

*Corynebacterium pseudotuberculosis* (CPS) is an important bacterial animal pathogen. CPS causes chronic, debilitating and currently incurable infectious diseases affecting a wide range of livestock and wild herbivores including camelids worldwide. Belonging to the *Corynebacterium diphtheria*e complex, this pathogen can also infect humans. The classical characterization of CPS is typically based on the testing of nitrate reductase activity, separating the two biovars Equi and Ovis. However, more refined resolutions are required to unravel routes of infection. This was realized in our study by generating and analyzing whole genome sequencing (WGS) data. Using newly created core genome multilocus sequence typing (cgMLST) profiles we were the first to discover isolates grouping in a cluster adjacent to clusters formed by CPS biovar Equi isolates. This novel cluster includes CPS isolates from alpacas, llamas, camels and dromedaries, which are characterized by a lack of nitrate reductase activity as encountered in biovar Ovis. This is of special interest for molecular epidemiology. Nevertheless, these isolates bear the genes of the nitrate locus, which are characteristic of biovar Equi isolates. However, sequence analysis of the genes *narG* and *narH* of the nitrate locus revealed indels leading to frameshifts and inactivity of the enzymes involved in nitrate reduction. Interestingly, one CPS isolate originating from another lama with an insertion in the MFS transporter (*narT*) is adjacent to a cluster formed by ovine CPS isolates biovar Equi. Based on this knowledge, the combination of biochemical and PCR based molecular biological nitrate reductase detection can be used for a fast and uncomplicated classification of isolates in routine diagnostics in order to check the origin of camelid CPS isolates. Further analysis revealed that partial sequencing of the ABC transporter substrate binding protein (CP258_RS07935) is a powerful tool to assign the biovars and the novel genomovar.

## Introduction

*Corynebacterium pseudotuberculosis* (CPS) is a pathogenic bacterium that belongs to the *Corynebacterium diphtheriae* complex and causes severe infections in ruminant and camelid species worldwide [1–4]. Caseous lymphadenitis (CLA) is the most important disease in sheep and goats caused by this pathogen; it poses a global animal welfare issue in small ruminant livestock [5–7]. However, other animal species are also affected. In water buffaloes CPS infections cause edematous skin disease [8] and in cattle infections are characterized by ulcerative granulomatous lesions [9, 10] or mastitis [11]. In horses ulcerative lymphangitis (pigeon fever) is the predominant clinical sign [12, 13]. The disease also affects other species such as pigs, deer and many wild animals such as Iberian ibex (*Capra pyrenaica*), pronghorns (*Antilocapra americana*), elk (*Cervus elaphus canadensis*) and Arabian oryx (*Oryx leucoryx*) [4, 14, 15]. Among the susceptible animal species are also camelids, which develop CLA with symptoms that are comparable to infections in small ruminants, including fatal abscesses in superficial lymph nodes and inner organs [16–19]. The growing popularity of alpaca husbandry in Europe has led to an increasing population [20–22] and number of animals infected with CPS, inflicting significant losses [18, 23, 24]. What makes the situation worse is that there is thus far no reliable cure or remedy against this insidious bacterial infectious disease [19, 25]. Therefore, the primary focus of prevention and combating of CPS infections lies in preventive herd management measures. This includes the monitoring of herds and surveillance of the herd status, pre‐screening of animals and control of direct contact among susceptible animals, as well as epidemiological investigations [16, 18, 25]. This is especially important because CPS can also possibly infect humans, causing CLA like lesions after contact with infected animals [26].

Among camelids little is known about the source and transmission routes of this pathogen within and between herds. In this context, molecular epidemiology is of particular relevance for revealing the origin, transmission routes and reservoirs of pseudotuberculosis infections [16, 18, 27]. This means that it is important to characterize CPS isolates in order to gather information on the extent to which these pathogens are related to each other and to provide information on their origin and current spread. This is especially true for isolates originating from camelids, which are still underrepresented [28]. In contrast to small ruminants, *Corynebacterium pseudotuberculosis* biovar Ovis is not the sole pathogen responsible for CLA in camels. Other pathogens such as *C*. *pseudotuberculosis* biovar Equi, other corynebacteria, *Streptococcus agalactiae* and *Staphylococcus* spp. may be involved [29–32]. Thus, many coryneform bacteria other than *C*. *pseudotuberculosis* are associated with CLA in dromedaries which necessitate multiple identification steps [33]. Preliminary characterization and differentiation of CPS isolates relies roughly on their classification into the two biovars Ovis and Equi, as a first step. Biotyping provides indicative information for the allocation of the biovars to animal species [1, 34–37]. Further genetic characterization of CPS isolates using reliable, reproducible and accurate techniques like multilocus sequence typing (MLST) [38–40] and recently core genome MLST (cgMLST) based on whole genome sequence data have been increasingly employed with great success [16, 35, 39]. However, there are only few comparative studies available on CPS genomes comprising all relevant susceptible animals [36, 37, 39]. Currently, 126 CPS isolates originating from 19 countries and regions have been reported (status April 15, 2023) [16], including only three whole genome sequences from camelids. In detail, these involved a camel from the UK (no detailed information on the species available; biovar Equi) [41], a llama from the USA (biovar Ovis) [42] and an alpaca from China (biovar Equi) [16]. Thus, there is still a dire lack of genome sequence data and comparative genomic studies on CPS isolates originating from camelids [28]. The increasing importance of CLA infection in camelids makes investigations on the epidemiology of CPS isolates essential. In practice, an unanswered question remains as to the genetic relationship between the various CPS isolates found in different animal species and whether interspecies transmission takes place [16, 18]. In this respect, whole genome sequencing (WGS) has so far proven to be the tool of choice for molecular epidemiological studies, providing a complete scope of molecular data for molecular characterization and in-depth typing of bacteria [16, 27, 43, 44]. Presenting cgMLST results using a minimum spanning tree (MST) is one of the most helpful tools currently in use for visualizing the relationship between isolates [45, 46]. In this context, the gene-by-gene approach represented by cgMLST has currently evolved as the most suitable method for molecular epidemiological investigations [47, 48]. This is because the cgMLST analysis has proven to be reproducible, stable and suitable for outbreak detection and surveillance purposes due to its high discriminatory power and its compatibility with conventional MLST.

The large genetic difference between the two biovars Ovis and Equi [37] makes the usual application of single nucleotide polymorphism (SNP) analysis difficult, as this is frequently based on a reference sequence that has the highest possible genetic match to the isolates examined. To overcome this limitation, a core genome MLST scheme for CPS based on cgMLST analysis was developed for an extensive gene-by-gene comparison. Use of this scheme makes it possible to obtain current, previously lacking molecular data for CPS isolates originating from camelids and other animal species for the purpose of molecular epidemiological investigations. A further aim of this study is to compare whole genome sequences of this pathogen in order to reveal genes or partial gene sequences that would be suitable for serving as representatives for genotyping of CPS isolates based on small-scale Sanger DNA sequencing.

## Results

### *Corynebacterium pseudotuberculosis* isolates

A number of CPS whole genomes from various host animals can be found in publicly available databases. However, in contrast to isolates from sheep, goats and horses, data sets of isolates from camelids are still underrepresented, even though the number of animals suffering from CPS infections is increasing. We have therefore performed WGS analysis on a number of CPS isolates available to us from diseased camelids. The dromedaries, alpacas and lamas included in this study showed visible abscesses in the skin and superficial lymph nodes. At post mortem examination abscesses in internal organs such as lungs, liver, spleen and kidney were found as also previously described [17, 18]. We were able to obtain WG sequences from six alpacas, two llamas, three dromedaries and three camels (no detailed information on the species available). Additionally, sequence data from isolates of four sheep and three goats were generated using next-generation sequencing technology (Illumina and partially Oxford Nanopore sequencing technology). The six CPS isolates originating in alpacas included in this study lived in three alpaca herds, two of which had had contact through trade and breeding. Details on the alpacas and the CPS isolates obtained from these animals have been previously presented [18]. In addition to the sequence analysis focusing on the nitrate locus, conventional tests for the nitrate reductase were performed. No CPS isolates, except for four ovine strains (CVUAS 3337, CVUAS 3338, CVUAS 3357, CVUAS 3361), showed any nitrate reductase activity (Table 1).

**Table 1:** *C*. *pseudotuberculosis* isolates included in this study for whole genome sequencing (WGS).

Strain	Biovar	Sequence ID	Accession-Number (NCBI)	Animal Species	Country of origin	Collection date (Year)	CAMP *S*. *aureus*	CAMP *R*. *equi*	Nitrate reductase
CVUAS 4258.2	Ovis	llama-CVUAS_4258.2	SRR25660940	llama	GE	2021	pos.	pos.	neg.
CVUAS 2997	Ovis	goat-CVUAS_2997	SRR22859700	goat	GE	2003	pos.	pos.	neg.
CVUAS 32354	Ovis	goat-CVUAS_32354	SRR22859691	goat	GE	2020	pos.	pos.	neg.
CVUAS 32656	Ovis	alpaca-CVUAS_32656	CP115162	alpaca	GE	2020	pos.	pos.	neg.
CVUAS 32689	Ovis	alpaca-CVUAS_32689	CP115163	alpaca	GE	2020	pos.	pos.	neg.
CVUAS 32746	Ovis	alpaca-CVUAS_32746	SRR22859683	alpaca	GE	2020	pos.	pos.	neg.
CVUAS 32834.2	Ovis	alpaca-CVUAS_32834.2	SRR22859692	alpaca	GE	2020	pos.	pos.	neg.
CVUAS 32842	Ovis	alpaca-CVUAS_32842	SRR22859685	alpaca	GE	2020	pos.	pos.	neg.
CVUAS 33314	Ovis	alpaca-CVUAS_33314	SRR22859684	alpaca	GE	2021	pos.	pos.	neg.
CVUAS 3337	Equi	sheep-CVUAS_3337	SRR22859693	sheep	GE	2004	pos.	pos.	pos.
CVUAS 3338	Equi	sheep-CVUAS_3338	SRR22859686	sheep	GE	2005	pos.	pos.	pos.
CVUAS 3357	Equi	sheep-CVUAS_3357	SRR22859688	sheep	GE	2006	pos.	pos.	pos.
CVUAS 3361	Equi	sheep-CVUAS_3361	SRR22859694	sheep	GE	2006	pos.	pos.	pos.
CVUAS 4988	Ovis	goat-CVUAS_4988	SRR22859699	goat	GE	2007	pos.	pos.	neg.
CVUAS 5583.2	Ovis	dromedary-CVUAS_5583.2	CP115164	dromedary	GE	2015	pos.	pos.	neg.
CVUAS 34900	Ovis	dromedary-CVUAS_34900	SRR25660942	dromedary	NL	2022	pos.	pos.	neg.
CVUAS 34905	Ovis	dromedary-CVUAS_34905	SRR25660941	dromedary	NL	2020	pos.	pos.	neg.
LGL KL293	Ovis	camel-KL293	SRR22859698	camel	GE	2011	pos.	pos.	neg.
LGL KL302	Ovis	camel-KL302	SRR22859697	camel	GE	2011	pos.	pos.	neg.
LGL KL359	Ovis	camel-KL359	SRR22859696	camel	GE	2012	pos.	pos.	neg.
LHL 191000189	Ovis	llama-191000189	SRR22859695	llama	GE	2019	pos.	pos.	neg.

GE = Germany, NL = Netherlands, neg. = negative, pos. = positive

### Full genome sequencing

Illumina short-read sequencing was used to analyze the DNA obtained from the CPS isolates. In order to achieve the most complete sequences possible and to close possible gaps that could occur due to the use of a single sequencing technology, Oxford nanopore long-reads were additionally generated for three of the isolates (2 x alpaca, 1 x dromedary). For the latter datasets, a hybrid assembly was created by MilongA by combining the short- and long-reads, which finally led to a single contig and therefore a closed genome. All other short-read datasets were assembled to draft genomes using SPAdes. The assembled genomes, like all other data sets from the NCBI database, were checked via an Average Nucleotide Identity (ANI) analysis. This ensures that the isolates of all data used belong to the species *C*. *pseudotuberculosis*. The sequence data of the CPS reference genome of isolate MEX29 (NZ_CP016826.1) and the CPS type strain ATCC 19410 (NZ_CP021251.1) were included in the analysis using the pyani program as well as a control group of *Corynebacterium ulcerans* (809), *C*. *silvaticum* (PO100/5), *C*. *diphtheriae* (ISS3319), *C*. *belfantii* (FRC0043) and *C*. *rouxii* (FRC0190) (S1 and S2 Figs and S1 Table). Both ANIm and ANIb were used for testing. Consistently, with the exception of the latter controls, all isolates considered to be CPS were well above the 95% percentage identity limit, which is usually considered the cut-off value for species differentiation [49] (S1 Table). Interestingly, a pattern emerged in this analysis. Even though the quantitative differences are not great, the biovar Ovis isolates, predominantly from sheep and goats, form a group. This is distinct from the biovar Equi isolates from horses and water buffaloes. A third group, which mainly contains the isolates from the camelids sequenced as part of this study, is still distinct from the other two groups, but is closer to the datasets of the isolates with biovar Equi (S1 and S2 Figs). Remarkably, however, the biochemical test of nitrate reductase activity assigned the isolates from the camelids to the biovar Ovis (Table 1).

To get a closer look at the genetic relations of the isolates of different hosts, a single nucleotide polymorphism (SNP) analysis was planned as a subsequent analysis. Using the variant calling pipeline snippySnake, data records originating of raw reads of a consistent sequencing platform can be examined for their SNP and clustered using these [50]. A basic requirement for reliable analysis of this approach is the use of a reference sequence as basis for comparison, that has the highest possible genetic match with all isolates to be analyzed. This sequence can be selected automatically by the pipeline. However, this turned out to be a problem when using all the isolates sequenced in this study in addition to data sets of CPS isolates from different hosts loaded from the SRA. The large genetic differences recognized by the pipeline during the analysis did not allow a common reference to be determined that could be used for all, which would lead to the exclusion of entire groups of data sets. These groups corresponded to those that also showed the previously mentioned minor differences in genetic similarity in the ANI analysis. The difficulties encountered here were a further indication of genetic differences between the isolates with biovar Ovis/Equi and the isolates from the camelids.

To solve the problem, a method for comparing the isolates should be used that does not require a common reference. For this purpose, core genome MLST can be used as a high-resolution differentiation method. Here the genes occurring in as many individual representatives of a species as possible are compared in terms of their sequences. This set of gene sequences organized within a so-called scheme, usually referred to as the core genome, is the basis of this comparative analysis. Unfortunately, for *Corynebacterium pseudotuberculosis*, in contrast to many other species, there is still no scheme available for such an analysis. Therefore, an appropriate scheme was generated for this study and will be made available during the revision via the website www.cgMLST.org.

### Development of a core genome MLST analysis scheme for *C*. *pseudotuberculosis*

A total of 187 genomes were analyzed in this study. The animal species from which the isolates were obtained were as follows: seven alpacas, four camels, four cattle, three dromedaries, 51 goats, 55 horses, five humans, three llamas, 37 sheep, 13 waterbuffaloes, one wildebeest and four unknowns. Of these genomes, 166 were obtained from the NCBI RefSeq and SRA databases (S2 Table), while 21 were obtained from whole genome sequencing of the CPS isolates listed in Table 1. To determine *C*. *pseudotuberculosis*’s core genome, Ridom SeqSphere+ software utilized complete or draft genomes following a recently published approach for *S*. *marcescens* [51]. Among 2,062 genes annotated for the CPS reference genome of strain MEX29 (NZ_CP016826.1), which was the seed genome, 1,577 targets were identified for cgMLST as they were observed in more than 97% of all genomes employed (mean percentage of good targets was 99.73%). For accessory targets, 456 were selected and 29 were discarded (S3–S5 Tables).

### Evaluation of the *C*. *pseudotuberculosis* cgMLST scheme

The absence of documented outbreaks with available WGS datasets hampers the conventional verification of a cgMLST scheme for *C*. *pseudotuberculosis*. However, in the context of a clarified laboratory accident, two WGS datasets were published. These include the isolate used in the laboratory and the isolate obtained later from the accidentally infected employee [52, 53]. Our cgMLST scheme was able to directly link these datasets with zero allele differences (AD). In addition, our clustering of isolates on the basis of the cgMLST allelic profile is in agreement with a recently published phylogeny [28]. For further verification, a subset of the data previously analyzed by cgMLST was examined using SNP analysis. SNP analysis is independent of the selected cgMLST targets and covers nearly the complete available genomic sequence. A high concordance with the number of genetic differences found between the two methods can therefore justify the usability of cgMLST. A selection of 14 isolates from different horses showed a very high degree of consistency in the detectable sequence differences between cgMLST and SNP analysis (Table 2).

**Table 2:** Comparison of the number of genetic differences of isolates from horses by cgMLST and SNP analysis.

Isolate-ID	Distances to reference	
	AD (cgMLST)	SNP
*horse-NC_016932* *	0	0
horse-SRR16507871	4	5
horse-SRR16507873	4	5
horse-SRR16507872	4	6
horse-SRR16507874	4	5
horse-SRR16507863	4	6
horse-SRR16507865	5	7
horse-SRR16507869	5	6
horse-SRR16507864	5	8
horse-SRR16507878	7	8
horse-SRR16507882	13	19
horse-SRR16507862	17	30
horse-SRR16507858	20	32
horse-SRR16507855	129	219

* = reference; biovar Equi

This was also the case for 22 further sequences including the camelid data, which were observed within a cgMLST cluster alongside sequences from human or sheep isolates that were significantly different from the aforementioned cluster (Table 3). However, at present, without sufficient data, it is not possible to set a threshold that characterizes a cluster of isolates (in an outbreak) on the basis of the maximum tolerated AD alone. This will only be feasible in the future with further use of cgMLST analysis.

**Table 3:** Comparison of the number of genetic differences of several isolates by cgMLST and SNP analysis.

Isolate-ID	Distances to reference	
	AD (cgMLST)	SNP
*dromedary-CVUAS_5583*.*2* *	0	0
camel-KL_359	1	4
camel-KL_293	1	4
llama-CVUAS_4258.2	3	9
camel-KL_302	3	5
alpaca-CVUAS_32842	5	11
alpaca-CVUAS_33314	6	12
alpaca-CVUAS_32746	12	15
alpaca-CVUAS_32656	12	15
alpaca-CVUAS_32834.2	13	15
alpaca-CVUAS_32689	13	16
dromedary-CVUAS_34905	89	142
dromedary-CVUAS_34900	92	144
sheep-CVUAS_3361	373	722
sheep-CVUAS_3337	373	722
sheep-CVUAS_3357	373	722
sheep-CVUAS_3338	373	722
llama-191000189	387	735
waterbuffalo-SRR7825416	1463	20944
waterbuffalo-SRR7825419	1463	20935
sheep-SRR14765337	1496	23014
sheep-SRR14765338	1497	23015

* = reference; biovar Ovis

### cgMLST analysis of *C*. *pseudotuberculosis* genomes

All datasets used to generate the cgMLST scheme have been studied using the 1,577 targets containing scheme. The examined isolates represent all pertinent animal species affected by this infection, along with the two previously described biovars—Equi and Ovis. When presented using the cgMLST minimum spanning tree (MST), the isolates clustered into several distinct groups. Said groups consist of isolates from specific animal species and, to some degree, from specified countries of origin.

**Fig 1:**
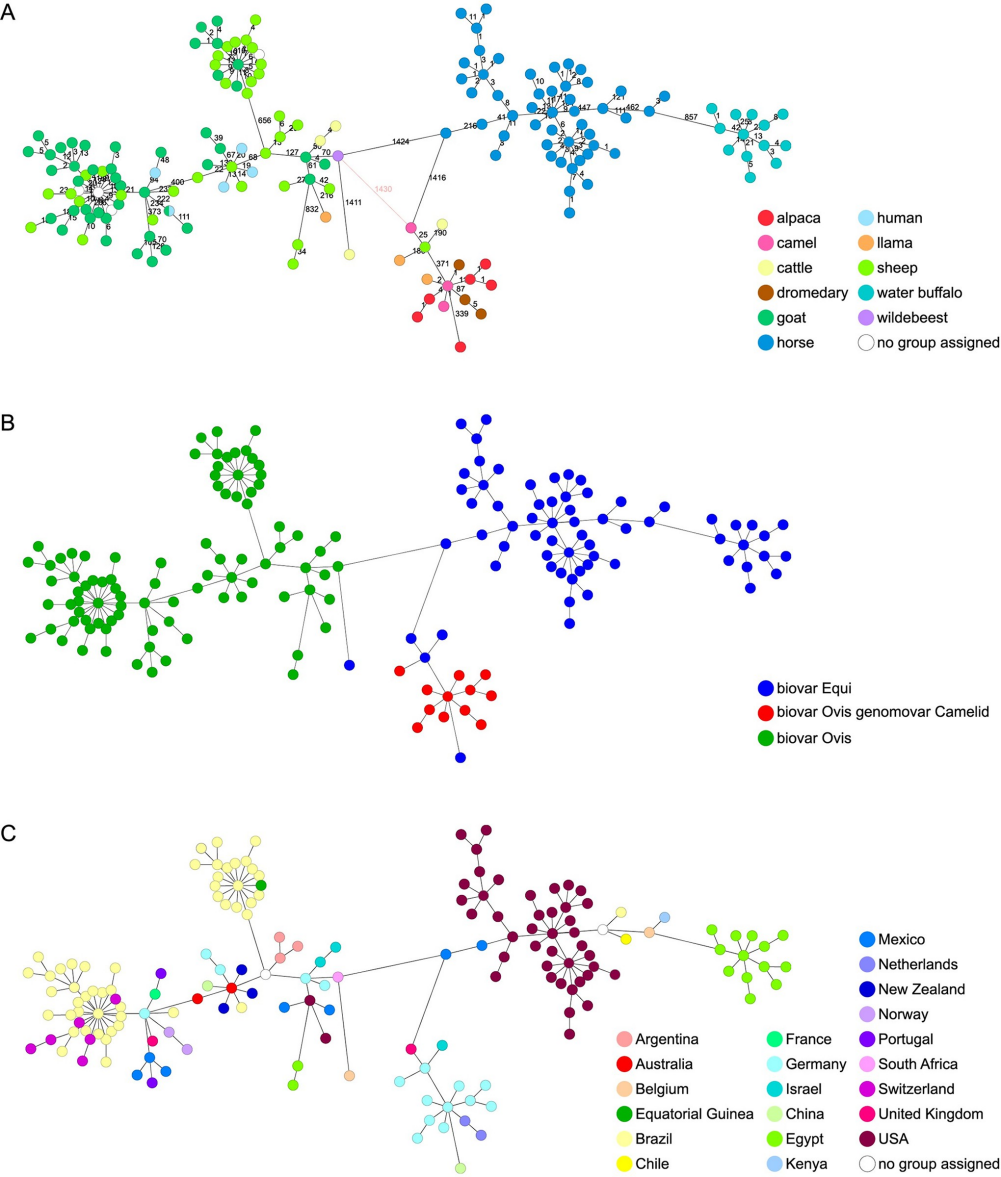
cgMLST result of 187 *C*. *pseudotuberculosis* genomes displayed graphically as minimum spanning tree (MST). 1,577 core genome targets were used. For distance calculation of the MST, the "pairwise ignore missing values" parameter was utilized. The distance between allelic genotypes is represented by the linkage between two MST nodes. The data on biotypes were retrieved from the NCBI GenBank and from our own investigations (21 CPS isolates). (A) Colors represent the different host animals of the isolates used. (B) Colors represent the three genotypes identified in this study, based on the detection of (intact) genes of the narKGHJI operon. (C) Colors represent the different geographic origins of the isolates.

From this perspective, host-specific groups in the MST include CPS isolates of horses mainly from the USA, and some from Brazil, Chile and Mexico (allele difference to other clusters >800) and water buffaloes from Egypt (allele difference of 957), which are clearly separated (all biovar Equi). In contrast, the CPS genomes of sheep and goats (all biovar Ovis) create a substantial cgMLST group that includes mixed animal species and is somewhat geographically defined. This group is distinct from the camelid, horse, and buffalo groups by 1,430 alleles (Fig 1). Interestingly, isolates acquired from humans have close associations with a group formed by CPS isolates from sheep from New Zealand, Australia, and Portugal (allele difference of 13–94). One further human isolate is identical to an isolate of a Norwegian strain collection (Fig 1). CPS isolates from a dromedary, a llama, alpacas and camels from Germany form a distinct cluster with a maximum distance of 11 AD. This cluster is clearly separated from the adjacent group by 371 AD, consisting of isolates from four German sheep (biovar Equi) and a UK camel (biovar Equi). Additionally, two CPS isolates from a German llama and an Israeli cow (both biovar Ovis) are located in this cluster as satellites (Fig 1 and S3 Fig). Furthermore, assessments that rely on host species show geographically specific groups and sub-groups to some degree (Fig 1).

### Biovar Equi and Ovis

As seen in the metadata on the genomes stored in the NCBI database, biovar Equi CPS isolates are found almost exclusively in horses and cattle. Our study confirmed that it is predominantly these species that are infected with this biovar, although we report here for the first time four biovar Equi isolates from sheep. Nevertheless, this appears to be an exception to the rule. The assignment to the biovar in general was carried out on the basis of our analysis of the genomic sequence. For this the presence of the nitrate reductase locus in the respective genome was tested. In contrast to biovar Ovis, the nitrate locus is found exclusively in the genome of isolates of biovar Equi [54, 55]. The reference genome of biovar Ovis strain MEX29, which was utilized to generate the cgMLST scheme, does not possess these genes, however. Consequently, a supplementary scheme was developed with Ridom SeqSphere+ using the well-described genome of biovar Equi CPS strain 258 [56], which comprised 17 genes of this locus as targets. Using this scheme, the examined isolates can be tested for the presence of the nitrate locus genes and any abnormalities within the gene sequence can be promptly identified. Whereas isolates from sheep, goats, horses and water buffaloes were consistently categorized as biovars based on the biochemical test and genetic analysis, the isolates from the camelids we sequenced showed different results. While these isolates did not show nitrate reductase activity and were accordingly categorized as biovar Ovis, the genomes of CPS isolates we sequenced from five alpacas, three camels, three dromedaries and one llama clearly carry the genes of the nitrate locus. Further analyses of the genes of the nitrate locus revealed a diverse pattern of genotypes revealing deletions within the gene encoding the alpha subunit of nitrate reductase *nar*G and the *nar*T gene, which encodes a transmembrane transporter. Furthermore, an insertion was detected in the gene of the ATP-binding cassette domain-containing protein (CP258_RS01950). However, this is not the only observable alteration. In contrast to these results, one CPS isolate from another llama (LHL 191000189), which had failed to reduce nitrate, revealed an insertion inside the *nar*H (nitrate reductase subunit beta) gene in addition to the already described insertion in CP258_RS01950. These mutations lead to frameshifts and, as a consequence, to a dysfunction of the nitrate reductase. While the effect of a defective nitrate reductase enzyme on the biochemical test is easy to understand, the influence of the defective ATP-binding protein CP258_RS01950 remains unclear. The indels described here were not found in the ovine and caprine CPS isolates sequenced in our study, which makes a systematic error in the generation of our own sequence data unlikely. Nevertheless, the affected region of the *nar*G gene of various isolates was checked with Sanger sequencing, and the SNP was confirmed on the basis of this independent analysis (S1 Data). In summary, an MST based on cgMLST analysis shows a unique group of isolates that differ significantly from those of the classic biovar Equi and Ovis. This group includes CPS isolates from alpacas, dromedaries, camels and a llama, characterized by a present but defective nitrate locus. Interestingly, a CPS biovar Equi isolate from a Chinese alpaca proved to be associated with this cluster, but with a great distance of 339 AD. In contrast, a further nitrate reductase negative isolate from a llama (LHL 191000189) bearing a mutation in the *nar*H gene is associated with an adjacent, but with 371 AD clearly separated, group formed by the four ovine biovar Equi isolates mentioned above (Fig 1A and S3 Fig).

The CPS isolates originating from sheep and goats belonging to the biovar Ovis cluster at a clear genetic distance from the biovar Equi CPS isolates. This holds also true for isolates from cattle originating from Belgium (biovar Equi) and Israel (biovar Ovis) that stand out from the other isolates (Fig 1).

### Genotyping based on single copy gene

The assignment of an isolate to the observed cgMLST groups can be helpful in determining the source of infection of an animal, especially when it comes to camelids. Here, the genotype can provide a clear indication of whether transmission from other animal species has taken place. For this reason, a simple method of genotyping was sought that does not require time-consuming and expensive WGS. The combination of the biochemical test for the presence of nitrate reductase and the PCR-based detection of a region within the nitrate locus, such as the *nar*G gene, can be used as a simple procedure for detecting the genomovar camelid. Isolates of Biovar Equi are positive in both tests due to the functional nitrate locus, isolates of Biovar Ovis are negative in each of them. Biovar Ovis genomovar camelid, on the other hand, shows no enzyme activity, but the gene can be detected by PCR (S4 Fig). This is a simple routine test to differentiate between the three variants. Sequencing can be used for a more detailed phylogenetic classification of an isolate.

**Fig 2:**
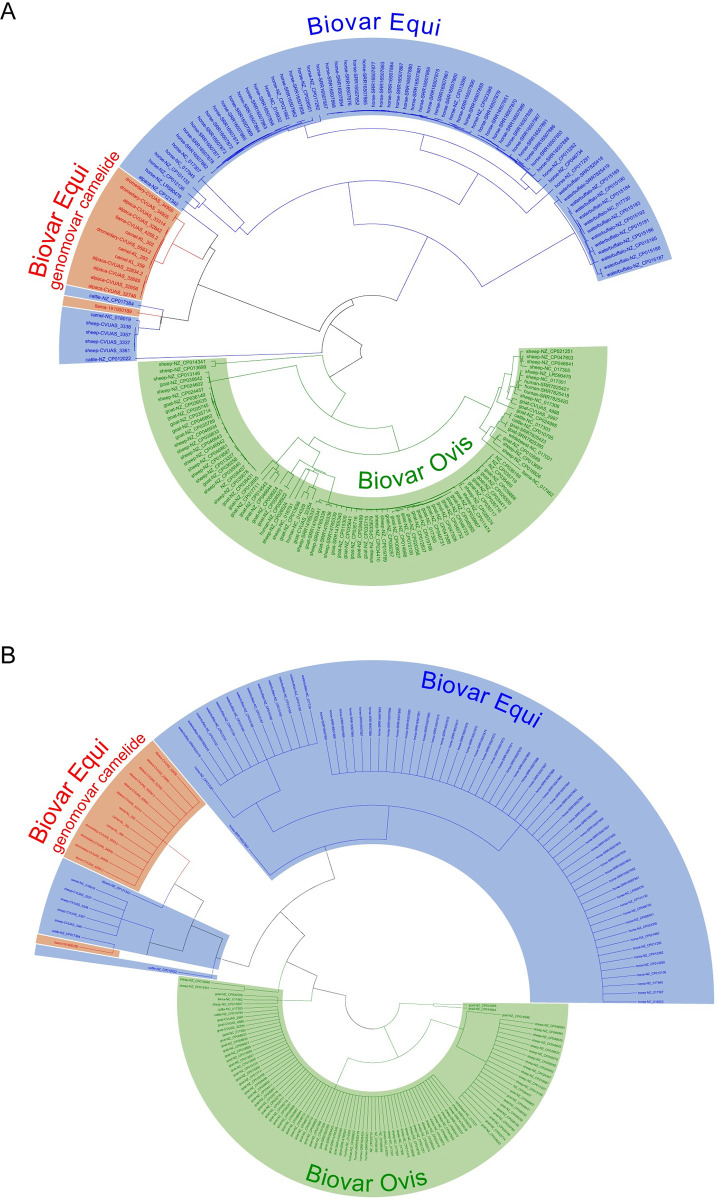
Phylogenetic trees of *C*. *pseudotuberculosis* genomic data. Phylogenetic trees of genomic data from of 187 *C*. *pseudotuberculosis* isolates. Biovars are represented by colors. Biovar Ovis in green, Biovar Equi with narKGHJI operon in blue. If no information on the biovar was included in the metadata, the biovar was determined based on the presence of the narKGHJI operon. The data sets with indel in the genes of the narKGHJI operon are marked in red and labeled as Biovar Ovis genomovar camelid. (A) Neighbor-joining tree based on cgMLST allelic profiles of 1,577 core genome targets. The "pairwise ignore missing values" parameter within Ridom SeqSphere+ was utilized for distance calculation. (B) DNA sequences of the ABC transporter substrate-binding protein CDS gene CpMEX29_RS07510, extracted from the whole genomes, were aligned by Clustal Omega. The tree was generated through the use of FastTree from the alignments, using an approximately-maximum-likelihood approach.

The data obtained during the cgMLST analysis were used with the tools of Ridom SeqSphere+ to select the PLD gene and a total of ten other single copy genes, due to presumably high differentiation power and a sequence length that can be decoded by Sanger sequencing. Only the sequences of the ABC transporter substrate binding protein CDS gene CpMEX29_RS07510 fulfilled the requirement to separate CPS isolates from camelids with mutations in the genes of the nitrate locus into a distinct cluster (Fig 2). The sequence data and labeling of all SNPs of the complete sequence is shown in S1 File.

### Phospholipase D and diphtheria toxin gene

Phospholipase D (PLD) represents the main virulence factor of CPS, which all isolates produce. All of the tested CPS isolates produced PLD, verified by inhibition of the hemolysis of *Staphylococcus aureus* and synergistic enhancing of the hemolysis with *Rhodococcus equi*. PLD activity was detectable by hemolysis interference for all CPS isolates (Table 1).

Screening for the presence of the diphtheria toxin gene in the genomes involved in this study showed negative results for all CPS isolates except for those originating from water buffaloes.

This analysis was carried out using another scheme created with the Ridom SeqSphere+ cgMLST Target Definer. The scheme includes the CPS virulence factors listed in the virulence factor database VFDB and the coding sequence (CDS) of the diphtheria toxin gene from *C*. *diphtheriae*. Further systematic differences in the analyzed isolates could not be detected with help of this scheme.

## Discussion

CPS is one of the most important causes of chronic, multiple diseases in different animal species, especially in small ruminants, but also increasingly in camelid species [1, 3, 4, 19, 57]. The growing popularity of keeping alpacas (*Lama pacos*) has led to a rapid increase in the population [21, 58] and exacerbation of CPS infections in this New World camelid species. Determination of whether these isolates are pathogens that have been transmitted from other animals such as sheep, goats or horses, or that were introduced into the herd when the alpacas were purchased, is crucial for the prevention of further infection. This knowledge can be achieved through detailed genetic analysis. However, there are few reports on in-depth characterization of CPS isolates originating from alpacas [16, 18]. Thus, the present study introduces a novel analytical tool and generates data for comparative characterization of whole genome sequences obtained from herbivores and camelid CPS isolates. This particular study focuses on molecular comparison of this pathogen originating from alpacas with isolates from other susceptible animals.

Among livestock, mostly small ruminants, but also other species such as horses, cattle and increasingly camelids are affected by CPS [2, 18, 19]. In-depth epidemiological studies are crucial for understanding the complexity of the interconnection between the host species involved and the geographical occurrence of CPS infections. For this purpose, whole genome sequencing has proven to be an ideally suited tool for molecular characterization of bacterial isolates [48, 59].

Analysis of 21 whole genome sequences of the CPS isolates included in this study revealed a genome size of about 2.4 Mb encoding >2,000 genes. This data is consistent with those previously reported for CPS isolates originating from humans [60] and various animal species such as sheep [61], goats [38], buffaloes [36, 62], horses [44, 63], cattle [11] and camelids such as alpacas [16], llamas [42], and dromedaries [41].

The WGS data of the sequenced CPS isolates yield mean average nucleotide identity (ANI) values of >98% for the reference genome. This unambiguously classifies the isolates as CPS, exceeding the threshold of 95% for taxonomic delineation of prokaryotic species [49, 64]. Therefore, classifying CPS biovar Equi and biovar Ovis as subspecies is not recommended, due to close genetic conformity [5, 15].

The cgMLST scheme presented here contains all annotated genes of the CPS isolate MEX29 that are present in more than 99% of all genome sequences available to us. As a percentage threshold, a presence in ≥ 95% of the analyzed genomes was requested. The procedure for creating a "soft defined core genome" MLST was followed, as can also be found in the BIGSdb Oxford and EnteroBase approach [65] and other publications [51]. As this procedure is based on a large number of genomes, it covers the variability of the species very well. In our study we used this newly created cgMLST scheme for creating a MST based on 1,577 targets for characterization of a comprehensive set of 187 CPS genomes originating from various animal species including camelids from different geographical origins.

Classification of CPS is still based on the phenotypic and genetic differentiation of CPS into the biotypes Equi and Ovis. This biotype scheme also indicates a degree of host specificity, particularly in relation to biovar Equi [26, 28]. Thus, studies generally resulted in the assignment of CPS isolates obtained from horses [40, 44], buffaloes and camelids to the biovar Equi [19, 36] and those from goats, sheep and also camelids to the biovar Ovis [17, 39, 40, 57]. A first description of the discovery of CPS isolates obtained from alpacas in China belonging to the biovar Equi was recently published [16]. In this context, Viana et al. [2018] stated in a different study that cattle and camels are the only species from which CPS biovar Ovis as well as biovar Equi have been isolated. Furthermore, the biovar Ovis isolates have never been found in horses or buffaloes and no natural infections have been detected with biovar Equi in sheep or goats [37]. However, we found biovar Equi isolates from four sheep in our strain collection. Due to the limited metadata available on the holdings, we could only speculate about a possible link between them.

In contrast, a complete genome sequence of a caprine CPS isolate (strain PA02) originating from Brazil has been deposited in the NCBI GenBank (CP015309.1) as biovar Equi but in the NCBI Bio Sample information (SAMN04867886, sample name: CpA12) as biovar Ovis. However, our analysis of the provided genome sequence revealed the absence of the narKGHJI operon encoding the nitrate reductase which is in conformity with biovar Ovis [54]. Consequently, Meng et al. (2023) concluded that the strain PA02 was mistakenly assigned to the biovar Equi [16].

The groups visibly separated in our cgMLST analysis confirm the genetic distinction between biovar Ovis and biovar Equi (Fig 1). In addition, molecular analysis of genes of the nitrate locus reveals an unexpectedly diverse pattern of clusters and biovars that goes far beyond classic biovar typing (S3 and S5 Figs). The isolates of the various camelids (alpaca, llama, dromedary, camel) that we analyzed could be assigned without exception to a new group that differs greatly from those of the traditional biovars Ovis and Equi. These isolates contain the nitrate locus, but with defects in the several genes that are necessary for the reduction of nitrate. This mainly involves the deletion of a nucleotide in the genes CP258_RS01950, *nar*G, and *nar*T. These mutations lead to a frameshift, resulting in incomplete gene products and thus to a negative result in the nitrate reduction test.

Interestingly, a further German CPS isolate (LHL 191000189) from a llama revealed a nucleotide deletion in the CP258_RS01950 gene and a nucleotide insertion in the *nar*H gene. Both of these in turn lead to the now known lack of enzyme reaction.

In fact, our investigations classify the camelid CPS isolates phenotypically as biovar Ovis, however, our genetic analysis using WGS revealed the presence of corrupted genes of the nitrate locus. Thus, in consideration of the ICNP [66], we propose subtyping these CPS isolates of the biovar Ovis into the novel genomovar Camelid. This is characterized by a negative nitrate reductase reaction and the presence of a nitrate locus in the genome which, however, contains non-functional genes, such as CP258_RS01950, *nar*G, *nar*H and *nar*T detected in this study.

The loss of function of the nitrate reductase is due to various indels leading to frameshifts and resulting in a discrepancy between biochemical testing and detection of the CP258_RS01950, *nar*G, *nar*H or *nar*T genes (by PCR). Such discrepancies are known and can be caused by variations in gene expression, amino-acid substitutions and mutations [67]. Frameshifts have also been identified in gene encoding proteins mainly related to transport mechanisms, suggesting a loss of function that is presumably not vital for survival [37]. For the clarification of such discordances caused by genetic variants, molecular tools have proven to be more reliable and sensitive than phenotypic tests [67].

From an evolutionary point of view and based on MLST results, biovar Ovis and biovar Equi strains are suggested to have a common evolutionary origin, although they form distinct genetic clusters [15, 35]. Moreover, it has been postulated that biovar Ovis evolved from biovar Equi through anagenesis, which has been defined as a progressive evolutionary change that takes place over time in a single genetic lineage [15, 37]. This observation is corroborated by a recent study on the genomes of CPS from horses, buffaloes, cows, a camel and an alpaca [28]. These genetic changes are driven by gene loss and mutations [15, 37]. Overall, CPS isolates have been described as genetically homogeneous and even exhibiting a high level of clonal homogeneity, despite their broad host spectrum [38, 39]. However, investigations of buffalo isolates revealed 48 unique genes located in the pathogenicity island PiCp12 also carrying the diphtheria toxin gene which might play a role in host adaptation [36]. In addition, Soares et al. [2013] [35] reported on a large deletion in the major pili *spa*A cluster in biovar Equi strains, in contrast to a complete *spa*A cluster in biovar Ovis CPS isolates. In view of virulence factors, we could see that CPS isolates of the biovar Equi and the novel camelid cluster lack three (*str*B, *spa*D, *sap*A) of the 23 virulence factors that are listed in the virulence factor database (VFDB) compared to biovar Ovis. Barauna et al. [2017] [27] have also reported an extensive repertoire of virulence factors and a greater variability of the genome of CPS biovar Equi based on comparative genomics analysis. Among the virulence factors of CPS, phospholipase D (PLD) is considered to be the most prominent and important. PLD is produced by all CPS isolates [1] and plays a crucial role in the initial stage of CPS infections, facilitating the spread of this pathogen into the host [68]. In contrast to PLD, the diphtheria toxin gene, which is characteristic of the members of the *C*. *diphtheria* complex, has thus far only been found in CPS isolates from water buffaloes. In accordance with previous studies [8, 36], we did not succeed in detecting the diphtheria toxin gene in any of the CPS isolates, except for those originating from buffaloes. Thus, the genetic variability is estimated to be greater than originally thought [27, 40]. Pathogenic bacteria that rely on living in restricted hosts and intracellularly in host tissues are known to undergo an evolutionary, adaptive reduction of the genome by gene mutations [69–71], loss of gene function and eventually gene loss [37]. This clock-like, stepwise loss of genes is an indication of a constant compulsion to eliminate genes in the sense of negative selection of redundant or not vital genes [72, 73]. We assume, therefore, that CPS not only experiences genetic development in one direction, but also parallel into different genotypes. In this respect, we were able to detect different genotypes characterized by different indels in the genes of the nitrate locus of camelid CPS isolates. In detail, camelid (alpaca, llama, dromedary, camel) CPS isolates bearing mutated CP258_RS01950, *nar*G, and *nar*T genes of the nitrate locus create a cluster clearly separate from a German llama with mutations in the CP258_RS01950 and *nar*H genes and from biovar Ovis and biovar Equi CPS isolates. Focusing on the host range, geographic distribution, and virulence characteristics, this phenomenon has also been described for *Mycobacterium* species of the tuberculosis complex [74–76]. The driving force for these genetic changes is a host-pathogen interaction resulting in co-evolution. As a result, studies have shown an association between animal-adapted lineages and different geographic regions and possible adaption to specific hosts [77]. In sum, considering the CPS biovar Equi or Ovis in animal species on the basis of WGS analysis, our study reveals the following correlations: CPS biovar Equi could be detected in isolates originating from horses, buffaloes, cattle, sheep, and camelids (alpaca, camel). CPS biovar Ovis isolates are not associated with horses or buffaloes. A novel, distinct and unique genomovar in camelid CPS isolates, characterized by indels in the CP258_RS01950, *nar*G and *nar*T gene or in the CP258_RS01950 and *nar*H gene, has only been verifiable in CPS isolates obtained from camelids thus far (alpaca, llama, dromedary, camel).

In our study we created a MST based on cgMLST analysis including genomes originating from various animal species and geographical regions. This MST contains a diversified pattern of clusters. In previous multi-locus sequence analysis (MLSA) studies only a few animal species and geographical areas were included and considered [1, 35–37, 39, 40]. Regarding camelids, only individual CPS isolates have been thoroughly characterized by WGS and included in comparative phylogenetic studies thus far [1, 16, 37, 40–42]. In our MST, the camelid CPS isolates obtained from German alpacas, a llama (CVUAS 4258.2), and German and Dutch dromedaries showed a novel cluster that revealed a close genetic relationship to each other (biovar Ovis with indels in the CP258_RS01950, *nar*G and *nar*T gene). Interestingly, this cluster is clearly separated from a Chinese alpaca isolate (biovar Equi). Furthermore, it separates from a group consisting of four sheep isolates (biovar Equi), a dromedary isolate (biovar Equi) and the isolate from a cow from Israel (biovar Equi). Another llama isolate from Germany (biovar Ovis) is more distantly genetically related with a deletion in the *nar*H gene and CP258_RS01950. In this vein, Viana and colleagues [2017, 2018] [36, 37] have already shown that isolates obtained from other hosts like camels might form additional host-specific sub-clusters and that divergent lineages can coexist. Further examples presented in this and previous studies show separate animal-specific geographic clusters formed by equine isolates from the USA and buffaloes from Egypt, respectively (Fig 1C). In contrast, one horse each from Belgium and Kenya (both biovar Equi) as well as two cows from Israel (biovar Ovis) were found in a common cluster [1, 36]. Thus, Bernardes and colleagues [2021] [1] concluded that geographical location seems to play an important role in comparative pan-genomic analysis.

With regard to CPS isolates originating in goats and sheep belonging to the biovar Ovis we see several clusters and sub-clusters dominated by geographical regions and animal species. Going beyond the cluster at sub-cluster level, this relationship becomes even clearer. We assume that parallel developed CPS strains and genetic divergence is due to co-evolution leading to different genotypes within the biovars.

Overall, the suitability of MLST studies for distinguishing between bacterial isolates of different geographic origin and even the same outbreak has also been previously reported [35, 39, 45]. The formation of clonal complexes of CPS isolates in dependence of the affected host and geographic origin has also been observed using ERIC-PCR [78]. The reason for these correlations is seen in the exchange of and contact between animals from different herds, regions and countries for breeding and trading purposes [16, 78]. This has been proven by the finding of genetically very similar CPS isolates from alpacas living in geographically differently localized herds, but having contact through trade or mating [18]. These CPS isolates revealed a minor genetic distance of 1–17 alleles. The results visible in the MST based on cgMLST for molecular biotyping also suggest that the transmission of CPS across species depends on the husbandry systems currently in use. While sheep and goat husbandry is characterized by close intra- and inter-species contact, camelids, horses and water buffaloes are mainly kept separate from other animal species. It is important to note that while a transfer of the pathogen between different animal species is possible, the available data suggests that such transfers are not common [35, 36, 79]. However, the human CPS isolates revealed the same biovar as the animal species with which they formed clusters. This assumption is supported through our MST by the close clustering of CPS isolates originating from humans and animals (goats and sheep). Infections have been attributed to occupational exposure to infected animals, environmental contamination [80], consumption of raw milk [81] or in a rare case contact to bacterial cultures [53]. Thus, it can be assumed that infections in humans are caused by close contact with infected animals or with contaminated animal products [26].

An easy way to recognize the genomovar Camelid is to combine the detection methods of nitrate reductase, i.e. the biochemical test for the enzyme and the detection of the encoding gene by PCR. However, if the highest discriminatory power is desired in the MLST analysis, WGS can facilitate the selection of suitable genes [40]. Those genes might be used for effective phylogenetic studies using conventional MLST or for the sequencing of single genes, which is also feasible in less specialized molecular routine laboratories [34, 39, 40, 45].

Following the study by El-Sebay et al. (2021) [34], we included the PLD gene sequence, as well as in a further step selected ten genes, in our study as possible candidates for the phylogenetic separation of camelid genotypes. The analysis of the gene sequence should generate a comparable grouping of isolates as observed in the MST based on the cgMLST. Although the 187 aligned PLD sequences showed a sequence heterogeneity of 3.6%, comparative analysis of these sequences were only able to reflect the main clusters created by the cgMLST based MST. In a previous study El-Sebay et al. (2021) [34] used sequences of the PLD gene to differentiate between CPS biovar Equi and biovar Ovis isolates from buffalo, cattle, sheep and goat, based on a sequence heterogeneity of 1.3%. Of the ten selected genes in this study, the ABC transporter substrate-binding protein gene (CP258_RS07935, 1509 bp) shows a sequence heterogeneity of 2.7% and proved to be better suited for a highly resolving differentiation of CPS isolates originating from camelids than the PLD gene. Sequences of this gene are able to separate camelid CPS isolates into different branches, in accordance with the clustering of the cgMLST based MST. Comparative results have been previously reported for selected genes of CPS, i.e. *dna*K or *asp*T, which represent genes with the most variability among those used for MLSA [39, 40]. Using amplicon sequencing, single-copy protein-coding genes have also been used to characterize sequence-discrete populations. For this hypervariable gene regions of about 200–550 bp were used [82].

## Conclusion

In conclusion, this is the first comprehensive comparative cgMLST study on CPS isolates suggesting a novel cluster, genomovar Camelid, including isolates originating from European camelids. The use of cgMLST allelic profiles has improved our knowledge of molecular epidemiology, which depends on the phylogeography of CPS (correlation of animal species and regional origin). In addition, we could show that conventional nitrate reductase testing in combination with detection of the *narG* gene by PCR is able to detect this novel genomovar. For more sophisticated analysis we propose a gene (ABC transporter substrate-binding protein) whose sequences decoded by feasible Sanger sequencing have proven to be suitable for serving as representative of cgMLST genotyping of CPS isolates in routine diagnostics.

Further studies are needed to monitor the spread of CPS isolates obtained from camelids and to gain knowledge about their pathogenic potential. For this purpose, WGS analysis has been shown to be a fundamental tool.

## Material and methods

### Ethics statement

The study was not submitted to any committee. The experiments do not require the approval of committees, as they involve genome sequencing and bacteriological testing of previously isolated bacterial strains. This means, the samples from the animals included in this study were exclusively taken for the purpose of regular monitoring on *C*. *pseudotuberculosis* during routine pathological diagnostic investigations of perished animals in state veterinary investigative agencies. Evaluation of the data occurred retrospectively. The samples were not taken with the aim of conducting this study. All patient owner records/information was anonymized and de-identified prior to analysis.

### Isolation, bacterial culture and characterization of CPS Isolates

CPS strains were previously isolated from abscesses of infected animals (Table 1). The isolates from alpacas came from a previous study and information regarding these isolates has been published [18]. For all types of analyses, the isolates were cultured aerobically on Columbia agar with 5% sheep blood (CSA, BD, Heidelberg, Germany) for 24–48 hours at 37°C. Corynebacterial cultures were identified at the species level using MALDI-TOF MS analysis (matrix-assisted laser desorption/ionization mass spectrometry; Bruker Daltonics, Bremen, Germany), employing the MALDI‐Biotyper System (Biotyper software Version 3.1, Bruker Daltonics). This was expanded by an in-house database [83] and updated with data obtained from *C*. *silvaticum*, *C*. *ulcerans*, and CPS [84]. The CPS specimens underwent a nitrate reduction test, in compliance with standard protocol [85], utilizing the CPS strain ATCC 43924^TM^ as a positive control. Detection of phospholipase D (PLD) activity was performed on the basis of hemolysis interference of the CPS isolates with *Staphylococcus aureus* (ATCC 25923T) and *Rhodococcus equi* (ATCC 33701T) on CSA agar [86].

### DNA extraction and whole genome sequencing

For short-read sequencing, DNA was extracted after bacterial culture on 5% sheep blood agar plates, using the DNeasy Blood & Tissue Kit (Qiagen, Hilden, Germany) in accordance with the manufacturer’s instructions. WGS was performed on a NovaSeq 6000 (S4 Reagent Kit v1.5) with 2x100 or 2x150 bp paired-end reads subsequent to Nextera XT library preparation (both Illumina, San Diego, USA), using 1 ng of genomic DNA. Library preparation and sequencing were done by CeGaT GmbH (Tübingen, Germany). For long-read sequencing the DNA was extracted using the MagAttract HMW DNA Kit (Qiagen, Hilden, Germany) in accordance with the manufacturer’s instructions. WGS was performed using Flongle Flow cells (FLO-FLG001), coupled with the Rapid Sequencing Kit (all Oxford Nanopore Technologies, Oxford, UK) according to the ONT protocols (Rapid Sequencing Kit SQK-RAD004; Flow cell priming Kit EXP-FLP002; Flongle Sequencing Expansion EXP-FSE001). The MinION Mk1B sequencing device MIN-101B was utilized together with the Flongle Adapter ADP-FLG001. Basecalling was performed using the “High-accuracy basecalling” model within the MinKNOW v. 21.06.0 software, with a minimum read filtering qscore of 9. The entire collection of WGS raw data is accessible at the NCBI sequence read archive (SRA) via https://www.ncbi.nlm.nih.gov/sra in the BioProject PRJNA914889.

### Creation of a (soft) core genome scheme for CPS

To create the cgMLST scheme, the previously described procedure for defining a "soft" core genome was applied [51]. In brief, to obtain a comprehensive collection for target scheme definition we downloaded publicly available whole genome sequence datasets for CPS (as of 13.11.2023) from the NCBI RefSeq genome database (n = 115 complete genome data sets in fasta format) and the sequence read archive (SRA, n = 55 data sets as fastq files). Only Illumina paired-end datasets from the SRA were used to maintain compatibility with SNP analysis software, which should be used afterwards to verify the cgMLST scheme. The data from the SRA as well as 21 self-generated data sets based on Illumina WGS paired-reads were assembled with the AQUAMIS pipeline (own Data with v. 1.3.7 using SPAdes v. 3.14.1, later downloaded SRA with v. 1.4.0 using SPAdes v. 3.15.5;) using default settings [87]. Three supplementary sequences were generated by performing a hybrid assembly of Illumina short-read and ONT long-read data using MilongA (https://gitlab.com/bfr_bioinformatics/milonga; default settings with the flye assembler v. 2.9-b1768). This pipeline entails the QC analysis of the raw reads, e.g. contamination checks and quality trimming. Removal of a set of data downloaded from the SRA and three genomes from the NCBI was necessary due to quality concerns. To ensure representation, the sequences were checked to include both biovars and as many different host species as possible. To ensure correct assignment to the species, an average nucleotide identity analysis using pyani was conducted. All records exceeded the identity value of ≥ 95% to the *C*. *pseudotuberulosis* type strain ATCC 19410 (NZ_CP021251.1) and were therefore retained. A total of 187 datasets formed the genome collection for defining cgMLST (S2 Table). The publicly available NCBI reference genome of *C*. *pseudotuberulosis* biovar Ovis strain MEX29 (NZ_CP016826.1) was selected as the seed genome, containing 2,062 genes. A preliminary target list for cgMLST was created using the cgMLST Target Definer tool (v. 1.5 with default parameters) within the Ridom SeqSphere+ software v. 9.0.10 (client). The list included all genes from the seed genome that did not contain internal stop codons, were not homologous, and did not overlap with other genes.

This preliminary target list was subsequently employed to analyze all the selected datasets for soft cgMLST typing scheme definition. A total of 1,577 targets from the preliminary list were utilized in the final cgMLST typing scheme as each was present in at least 97% of datasets (S3 Table). Meanwhile, 456 targets were transferred to a list of accessory targets because they were not found frequently enough in the genome data of the used strains (S4 Table). 29 targets were discarded as they did not fulfill the above-mentioned requirements and, for example, contained internal stop codons or overlapped with other genes (S5 Table). With the assistance of the Ridom SeqSphere+ cgMLST Target Definer tool, we have generated an additional task template. This template was based on the annotated genome of biovar Equi strain 258 (NC_017945.3) and designed to contain the exclusive coding regions for the nitrate reductase operon in biovar Equi (S6 Table). Furthermore, the Ridom SeqSphere+ cgMLST Target Definer was once again utilized to create a scheme (S7 Table) comprising the CPS virulence factors listed in the VFDB (http://www.mgc.ac.cn/cgi-bin/VFs/compvfs.cgi). The factors defined there are based on the annotation of CPS strain FRC41’s (NC_014329) genome. The factor CPFRC_RS09580* (*spa*C) could not be included in the scheme because it could not be extracted from the annotated genome deposited in NCBI. The sequence of the diphtheria toxin gene DIP_RS12515 from *C*. *diphtheriae* NCTC 13129 (NC_002935) was added to the scheme.

### Evaluation of the newly created core genome scheme

The evaluation of the novel cgMLST scheme was carried out in two ways. The classical method of evaluation, checking against known clusters, is difficult because there is very little available data in the literature. Dataset gleaned from an infection known to have occurred in the laboratory during an accident was therefore used [52, 53]. As an additional evaluation, a SNP analysis, which is independent of the cgMLST scheme, was performed on a selection of the data sets. The results were compared with those from the cgMLST analysis.

### Core genome analysis of CPS isolates

The previously described cgMLST scheme was used to characterize the selection of 187 CPS WGS datasets (S2 Table). This selection incorporates all the data used for the creation of the scheme (S2 Table). As previously stated, the WGS raw reads underwent processing by AQUAMIS, followed by importation of the assembled files in fasta format into Ridom SeqSphere+ and cgMLST typing with the default settings. The SeqSphere+ Software automatically called and assigned alleles for each target to ensure a unique nomenclature. The allelic profile of each strain was created from a combination of all alleles. This profile was then utilized to produce minimum spanning trees (MST) with the "pairwise ignore missing values" parameter utilized in distance calculation. The distance between allelic genotypes is represented by the linkage between two MST nodes. These distances were employed to ascertain whether the connected isolates could be assigned to the same group while remaining clearly distinct from other groups.

### Average nucleotide identity (ANI) analysis

All Whole Genome Sequencing (WGS) data assembled in this study were analyzed using the average nucleotide identity (ANI) technique for delineating species boundaries. The results confirmed the identity of species above the commonly used 95% identity threshold [49, 88]. As controls, the genomes were compared to the CPS reference genome of strain MEX29 (NZ_CP016826.1) as well as *C*. *belfantii* FRC0043 (GCF_900205605), *C*. *diphtheria* ISS3319 (NZ_CP0252091), *C*. *rouxii* FRC0190 (NZ_LR738855), *C*. *ulcerans* 809 (NC_017317) and *C*. *silvaticum* PO100/5 (NZ_CP021417). ANIm (MUMmer algorithm) and ANIb (BLASTN+ algorithm) analyses were performed with help of the pyani software v. 0.2.12 (https://pyani.readthedocs.io) [88].

### SNP variant calling

Analysis of single-nucleotide polymorphism (SNP) variant calling was performed within the pipeline snippySnake (v1.2.3; https://gitlab.com/bfr_bioinformatics/snippySnake) with the default settings. WGS data used were quality checked and trimmed by AQUAMIS. When analyzing the isolates from the camelids, the closed genome of sample CVUAS_5583.2 (CP115164), created by MilongA hybrid assembly was used as reference sequence. In the analysis of the isolates from the horses, however, the full genome of CPS strain 316 (NC_016932) taken from the NCBI database was used.

### PCR of *nar*G

After cultivation on 5% sheep blood agar plates, DNA from CPS colonies was prepared for PCR analysis by heat lysis followed by centrifugation. Supernatants were used as templates for amplification together with the PCR mastermix AccuStart™ II PCR SuperMix (VWR, Bruchsal, Germany). The following PCR program was used: 3 min at 94°C; 45x (20 sec at 94°C, 30 sec at 50°C, 90 sec at 72°C); 5 min 72°C. For *nar*G PCR, the primers CPS_narG-F (5´-GACTTGTCCAAGCGGTTCTCA -3´) and CPS_narG-R (5´-CACCGTTAGATACTCCCGACAT-3´), designed with Primer-BLAST [89], were used. To check the suitability of the template for PCR, a section of the PLD gene was also amplified in a parallel approach using the primers described by Sá et al. [68]. The PCR products were separated by agarose gel electrophoresis on a 2% gel and stained with Peqgreen (VWR, Bruchsal, Germany). The fluorescence was recorded with a Gel Doc XR+ Gel Documentation System (Bio-Rad Laboratories GmbH, Feldkirchen, Germany) using the automatic exposure control within the Image Lab Software to avoid oversaturation.

### Sanger sequencing of *nar*G

Bacterial DNA was extracted and purified using the Magnetic Bead Kit Mag-Bind® Total Pure NGS (VWR; Bruchsal; Germany) on a KingFisher Flex device (Thermo Scientific, Schwerte, Germany). For amplification of partial *nar*G gene sequences by PCR, the mastermix AccuStart™ II PCR SuperMix (VWR, Bruchsal, Germany) was used. The PCR product was sequenced on demand (Microsynth, Balgach, Switzerland) based on the Sanger DNA sequencing technique. The partial DNA sequence of the *nar*G gene was amplified using primers designed on the basis of sequence alignments of *nar*G gene sequences. The *nar*G gene sequences were obtained from a total of 90 CPS biovar Equi genomes originating from horses, water buffaloes, camels, alpacas, llamas, cattle, and sheep. Primer design was carried out using the Primer-BLAST (National Center for Biotechnology Information, https://www.ncbi.nlm.nih.gov/tools/primer-blast/). The PCR product (489 bp) was sequenced bidirectionally using the PCR primers listed above.

### Selection of single copy genes for genotyping

In order to find genes whose sequences could be used to group the isolates in a similar way to the cgMLST analysis, the targets (i.e. genes) listed in the Ridom SeqSphere+ comparison tables were searched manually. These tables were created as part of the comparative analysis of the various isolates using either the core or the accessory genome.

## Supporting information

S1 Raw images Uncropped and unadjusted original gel image presented as S4 Fig. PCR products of the amplification of the *nar*G or PLD gene.
(PDF)

S1 Fig ANIm heatmap of percentage identity for 187 *C*. *pseudotuberculosis* genomes. (PDF)

S2 Fig ANIb heatmap of percentage identity for 187 *C*. *pseudotuberculosis* genomes. (PDF)

S3 Fig Ridom SeqSphere+ cgMLST result of 187 *C*. *pseudotuberculosis* genomes. 1,577 core genome targets were used and displayed graphically as a minimum spanning tree (MST). For distance calculation of the MST, the "pairwise ignore missing values" parameter was utilized. The distance between allelic genotypes is represented by the linkage between two MST nodes. The animal species from which the isolates were obtained is included in the labeling (sequence-ID). The data on biotypes were retrieved from the NCBI GenBank and our own investigations (21 CPS isolates). If no information was available in the metadata, the biovar was assigned based on detection of genes of the narKGHJI operon encoding the nitrate reductase within whole genome sequences. These biovars are labeled with an asterix.
(SVG)

S4 Fig PCR products of the amplification of the *nar*G or PLD gene. Gene-specific primers were used to amplify parts of the *nar*G and PLD genes. The latter was used as a control. Genomic DNA of the indicated isolates served as template. The PCR products were separated by agarose gel electrophoresis in a 2% agarose gel. The DNA was stained with peqGREEN.
(TIF)

S5 Fig Neighbor joining tree of 187 *C*. *pseudotuberculosis* cgMLST allelic profiles. Metadata of the isolates are included as well as details of the nitrate locus genes. The indication “? (not found)” = gene not found; “? (failed)” = gene found, but with errors (mostly indel) in the sequence; “numbers” = numbers represent the allelic type of the gene found. If no information was available in the metadata, the biovar was assigned based on detection of genes of the narKGHJI operon encoding the nitrate reductase within whole genome sequences. These biovars are labeled with an asterix.
(SVG)

S6 Fig Phylogenetic tree of genomic data from of 187 *C*. *pseudotuberculosis* isolates. Biovars are represented by colors. If no information on the biovar was included in the metadata, the biovar was determined based on the presence of the narKGHJI operon. The data sets with indel in the genes of the narKGHJI operon are marked in red and labeled as Biovar Ovis genomovar camelid. DNA sequences of the ABC transporter substrate-binding protein CDS gene CpMEX29_RS07510, extracted from the whole genomes, were aligned by Clustal Omega v. 1.2.2 using default settings within the Geneious Prime Software (v. 2024.0.7). The corresponding gene sequence of *C*. *silvaticum* P0100/5 (NZ_CP021417; locus taq CBE74_RS21375) was used as outgroup. The tree from the alignments was generated through the use of the Geneious Tree Builder, using Tamura-Nei as genetic distance model and neighbor-joining as tree build method. The consensus tree was generated by resampling using bootstrap method and default settings.
(SVG)

S1 Table Results of pyani analysis (ANIm and ANIb) as distance matrix. (XLSX)

S2 Table List of *C*. *pseudotuberculosis* strains/sequences including the NCBI accession numbers and biovars used for cgMLST scheme creation and analysis. If no information was available in the metadata, the biovar was assigned based on detection of genes of the narKGHJI operon encoding the nitrate reductase within whole genome sequences. These biovars are labeled with an asterix.
(XLSX)

S3 Table List of genes used for cgMLST scheme as targets with reference to *C*. *pseudotuberculosis* strain MEX29. (XLSX)

S4 Table List of genes used for cgMLST accessory scheme with reference to *C*. *pseudotuberculosis* strain MEX29. (XLSX)

S5 Table List of discarded genes within the cgMLST scheme creation. (XLSX)

S6 Table List of genes used for cgMLST nitrate locus specific scheme with reference to *C*. *pseudotuberculosis* strain 258. (XLSX)

S7 Table List of genes taken from the VFDB and listed there as virulence factors of *C*. *pseudotuberculosis*. (XLSX)

S1 Data *nar*G Sanger vs WGS: SNP within the *nar*G gene sequences of different *C*. *pseudotuberculosis* isolates. (DOCX)

S1 File DNA sequence of the ABC transporter substrate-binding protein CDS gene RS07935. SNP detected by alignment of the different isolates included in this study are labeled.
(DOCX)
