# Supplementary material for: Corynebacterium pseudotuberculosis: Whole genome sequencing reveals unforeseen and relevant genetic diversity in this pathogen
Source: PLoS One. 2024 Aug 26;19(8):e0309282. doi: 10.1371/journal.pone.0309282 (PMC11346948; doi:10.1371/journal.pone.0309282)
Supplement: S1 Raw images — PCR products of the amplification of the narG or PLD gene. (PDF) [file pone.0309282.s001.pdf]

Raw gel image, source for S4 Fig

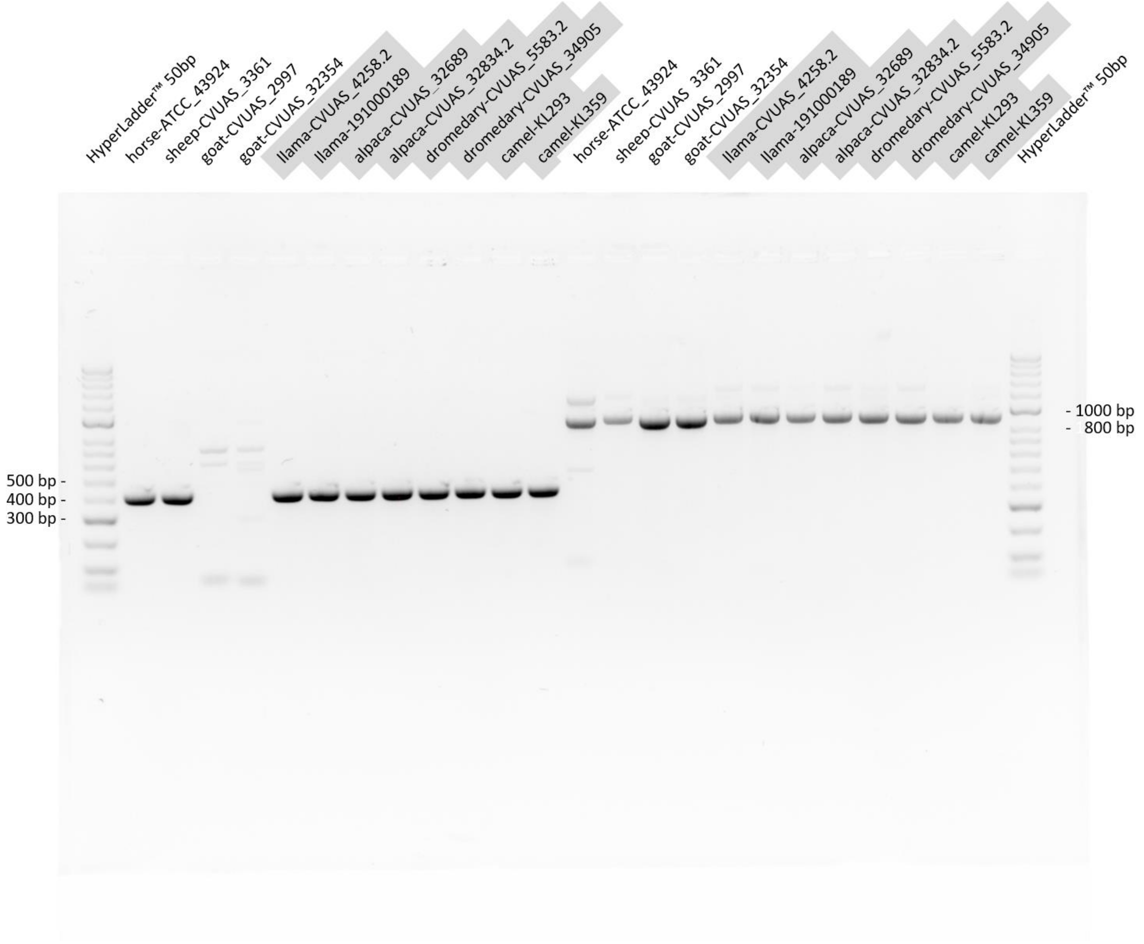

The PCR products were separated by agarose gel electrophoresis on a 2% gel and stained with Peqgreen (VWR, Bruchsal, Germany). The fluorescence was recorded with a Gel Doc XR+ Gel Documentation System (Bio-Rad Laboratories GmbH, Feldkirchen, Germany) using the automatic exposure control within the Image Lab Software to avoid oversaturation.
