# Supplementary figures and images for: Corynebacterium pseudotuberculosis: Whole genome sequencing reveals unforeseen and relevant genetic diversity in this pathogen
Source: PLoS One. 2024 Aug 26;19(8):e0309282. doi: 10.1371/journal.pone.0309282 (PMC11346948; doi:10.1371/journal.pone.0309282)

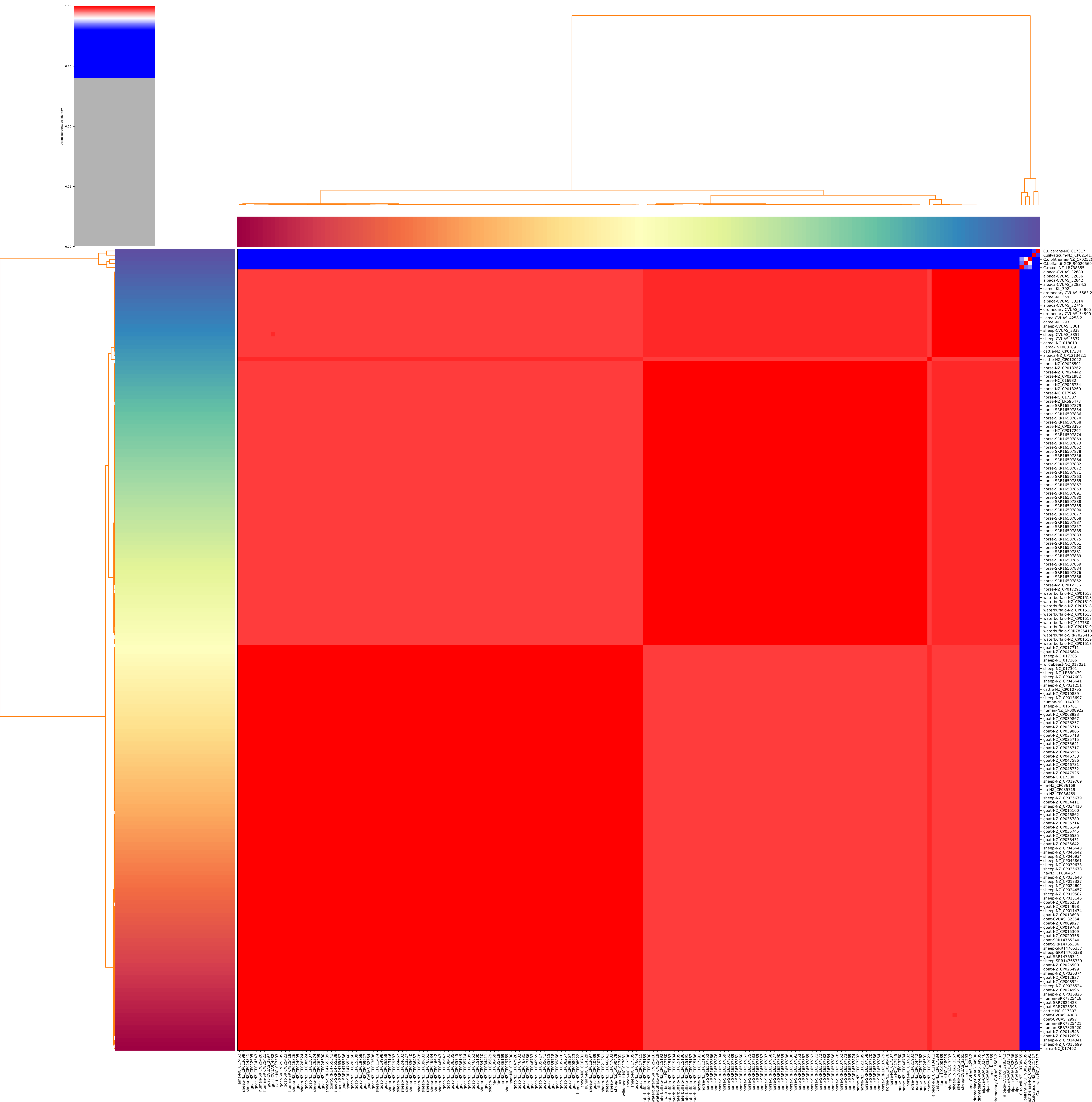

Supplement: S1 Fig — (PDF) [file pone.0309282.s002.pdf]

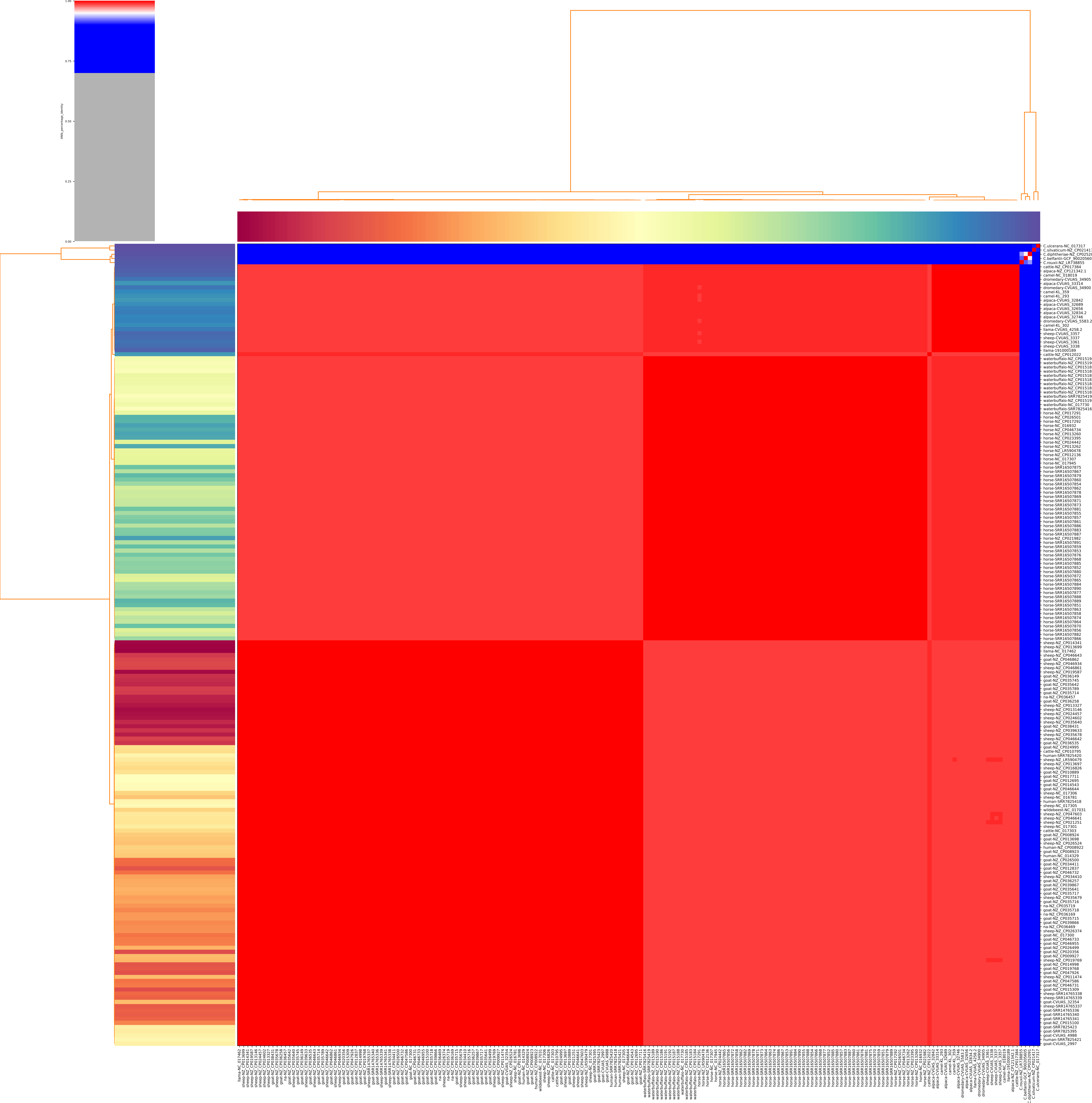

Supplement: S2 Fig — (PDF) [file pone.0309282.s003.pdf]

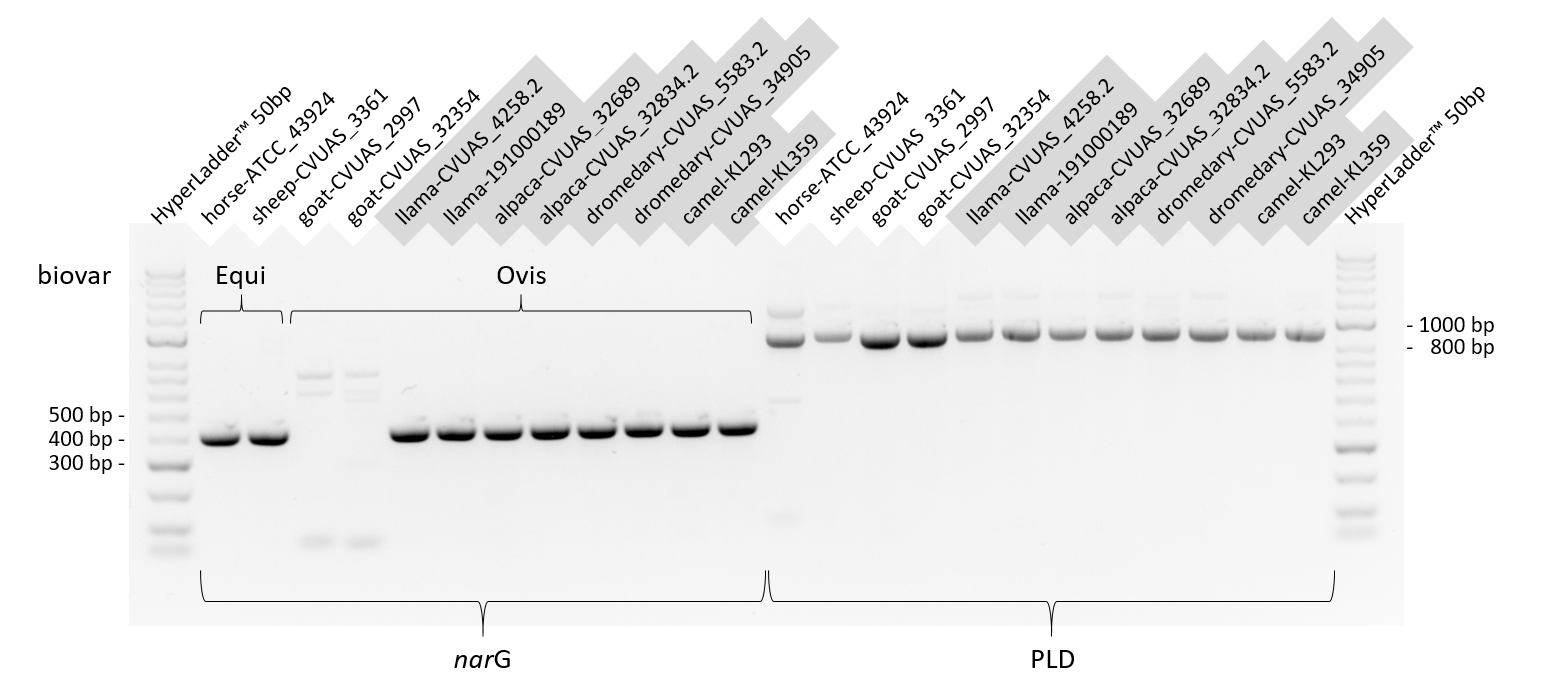

Supplement: S4 Fig — Gene-specific primers were used to amplify parts of the narG and PLD genes. The latter was used as a control. Genomic DNA of the indicated isolates served as template. The PCR products were separated by agarose gel electrophoresis in a 2% agarose gel. The DNA was stained with peqGREEN. (TIF) [file pone.0309282.s005.tif]
