# Supplementary material for: Corynebacterium pseudotuberculosis: Whole genome sequencing reveals unforeseen and relevant genetic diversity in this pathogen
Source: PLoS One. 2024 Aug 26;19(8):e0309282. doi: 10.1371/journal.pone.0309282 (PMC11346948; doi:10.1371/journal.pone.0309282)
Supplement: S1 Data — (DOCX) [file pone.0309282.s015.docx]

alpaca-CVUAS_32656 ovis WGS ATGTCATCTACTGACATGCACCCGTACCTGCACTCTTTCAATGCTGCTATTAACCCACCGTGGGAAGCACGCACAGATTT 2480

alpaca-CVUAS_32689 ovis WGS ATGTCATCTACTGACATGCACCCGTACCTGCACTCTTTCAATGCTGCTATTAACCCACCGTGGGAAGCACGCACAGATTT 2480

alpaca-CVUAS_32746 ovis WGS ATGTCATCTACTGACATGCACCCGTACCTGCACTCTTTCAATGCTGCTATTAACCCACCGTGGGAAGCACGCACAGATTT 2480

alpaca-CVUAS_32834.2 ovis WGS ATGTCATCTACTGACATGCACCCGTACCTGCACTCTTTCAATGCTGCTATTAACCCACCGTGGGAAGCACGCACAGATTT 2480

alpaca-CVUAS_32842 ovis Sanger --------------------------------------------------------------------------------

alpaca-CVUAS_32842 ovis WGS ATGTCATCTACTGACATGCACCCGTACCTGCACTCTTTCAATGCTGCTATTAACCCACCGTGGGAAGCACGCACAGATTT 2480

alpaca-CVUAS_33314 ovis WGS ATGTCATCTACTGACATGCACCCGTACCTGCACTCTTTCAATGCTGCTATTAACCCACCGTGGGAAGCACGCACAGATTT 2480

camel-KL_293 ovis WGS ATGTCATCTACTGACATGCACCCGTACCTGCACTCTTTCAATGCTGCTATTAACCCACCGTGGGAAGCACGCACAGATTT 2480

camel-KL_302 ovis WGS ATGTCATCTACTGACATGCACCCGTACCTGCACTCTTTCAATGCTGCTATTAACCCACCGTGGGAAGCACGCACAGATTT 2480

camel-KL_359 ovis WGS ATGTCATCTACTGACATGCACCCGTACCTGCACTCTTTCAATGCTGCTATTAACCCACCGTGGGAAGCACGCACAGATTT 2480

dromedary-CVUAS_5583.2 ovis WGS ATGTCATCTACTGACATGCACCCGTACCTGCACTCTTTCAATGCTGCTATTAACCCACCGTGGGAAGCACGCACAGATTT 2480

horse-ATCC43924 equi Sanger --------------------------------------------------------------------------------

horse-NC_017945 equi WGS ATGTCATCTACTGACATGCACCCGTACTTGCACTCTTTCAATGCTGCTATTAACCCGCCATGGGAAGCACGCACAGATTT 2480

llama-191000189 ovis Sanger --------------------------------------------------------------------------------

llama-191000189 ovis WGS ATGTCATCTACTGACATGCACCCGTACCTGCACTCTTTCAATGCTGCTATTAACCCACCGTGGGAAGCACGCACAGATTT 2480

llama-CVUAS_4258.2 ovis Sanger --------------------------------------------------------------------------------

llama-CVUAS_4258.2 ovis WGS ATGTCATCTACTGACATGCACCCGTACCTGCACTCTTTCAATGCTGCTATTAACCCACCGTGGGAAGCACGCACAGATTT 2480

sheep-CVUAS_3337 equi WGS ATGTCATCTACTGACATGCACCCGTACCTGCACTCTTTCAATGCTGCTATTAACCCACCGTGGGAAGCACGCACAGATTT 2480

sheep-CVUAS_3338 equi WGS ATGTCATCTACTGACATGCACCCGTACCTGCACTCTTTCAATGCTGCTATTAACCCACCGTGGGAAGCACGCACAGATTT 2480

sheep-CVUAS_3357 equi Sanger --------------------------------------------------------------------------------

sheep-CVUAS_3357 equi WGS ATGTCATCTACTGACATGCACCCGTACCTGCACTCTTTCAATGCTGCTATTAACCCACCGTGGGAAGCACGCACAGATTT 2480

sheep-CVUAS_3361 equi WGS ATGTCATCTACTGACATGCACCCGTACCTGCACTCTTTCAATGCTGCTATTAACCCACCGTGGGAAGCACGCACAGATTT 2480

alpaca-CVUAS_32656 ovis WGS TGAGGTTTTCCGCGACTTGTCCAAGCGGTTCTCAGAAATGGCAGTTACGTGGTTGGGCGTACAACACGATGTTGTCACAA 2560

alpaca-CVUAS_32689 ovis WGS TGAGGTTTTCCGCGACTTGTCCAAGCGGTTCTCAGAAATGGCAGTTACGTGGTTGGGCGTACAACACGATGTTGTCACAA 2560

alpaca-CVUAS_32746 ovis WGS TGAGGTTTTCCGCGACTTGTCCAAGCGGTTCTCAGAAATGGCAGTTACGTGGTTGGGCGTACAACACGATGTTGTCACAA 2560

alpaca-CVUAS_32834.2 ovis WGS TGAGGTTTTCCGCGACTTGTCCAAGCGGTTCTCAGAAATGGCAGTTACGTGGTTGGGCGTACAACACGATGTTGTCACAA 2560

alpaca-CVUAS_32842 ovis Sanger ----------------------------TTCTCAGAAATGGCAGTTACGTGGTTGGGCGTACAACACGATGTTGTCACAA 52

alpaca-CVUAS_32842 ovis WGS TGAGGTTTTCCGCGACTTGTCCAAGCGGTTCTCAGAAATGGCAGTTACGTGGTTGGGCGTACAACACGATGTTGTCACAA 2560

alpaca-CVUAS_33314 ovis WGS TGAGGTTTTCCGCGACTTGTCCAAGCGGTTCTCAGAAATGGCAGTTACGTGGTTGGGCGTACAACACGATGTTGTCACAA 2560

camel-KL_293 ovis WGS TGAGGTTTTCCGCGACTTGTCCAAGCGGTTCTCAGAAATGGCAGTTACGTGGTTGGGCGTACAACACGATGTTGTCACAA 2560

camel-KL_302 ovis WGS TGAGGTTTTCCGCGACTTGTCCAAGCGGTTCTCAGAAATGGCAGTTACGTGGTTGGGCGTACAACACGATGTTGTCACAA 2560

camel-KL_359 ovis WGS TGAGGTTTTCCGCGACTTGTCCAAGCGGTTCTCAGAAATGGCAGTTACGTGGTTGGGCGTACAACACGATGTTGTCACAA 2560

dromedary-CVUAS_5583.2 ovis WGS TGAGGTTTTCCGCGACTTGTCCAAGCGGTTCTCAGAAATGGCAGTTACGTGGTTGGGCGTACAACACGATGTTGTCACAA 2560

horse-ATCC43924 equi Sanger ----------------------------TTCTCAGAAATGGCAGTTACGTGGTTGGGCGTACAACACGATGTTGTCACAA 52

horse-NC_017945 equi WGS TGAGGTTTTCCGCGACTTGTCCAAGCGGTTCTCAGAAATGGCAGTTACGTGGTTGGGCGTACAACACGATGTTGTCACAA 2560

llama-191000189 ovis Sanger ----------------------------TTCTCAGAAATGGCAGTTACGTGGTTGGGCGTACAACACGATGTTGTCACAA 52

llama-191000189 ovis WGS TGAGGTTTTCCGCGACTTGTCCAAGCGGTTCTCAGAAATGGCAGTTACGTGGTTGGGCGTACAACACGATGTTGTCACAA 2560

llama-CVUAS_4258.2 ovis Sanger ----------------------------TTCTCAGAAATGGCAGTTACGTGGTTGGGCGTACAACACGATGTTGTCACAA 52

llama-CVUAS_4258.2 ovis WGS TGAGGTTTTCCGCGACTTGTCCAAGCGGTTCTCAGAAATGGCAGTTACGTGGTTGGGCGTACAACACGATGTTGTCACAA 2560

sheep-CVUAS_3337 equi WGS TGAGGTTTTCCGCGACTTGTCCAAGCGGTTCTCAGAAATGGCAGTTACGTGGTTGGGCGTACAACACGATGTTGTCACAA 2560

sheep-CVUAS_3338 equi WGS TGAGGTTTTCCGCGACTTGTCCAAGCGGTTCTCAGAAATGGCAGTTACGTGGTTGGGCGTACAACACGATGTTGTCACAA 2560

sheep-CVUAS_3357 equi Sanger ----------------------------TTCTCAGAAATGGCAGTTACGTGGTTGGGCGTACAACACGATGTTGTCACAA 52

sheep-CVUAS_3357 equi WGS TGAGGTTTTCCGCGACTTGTCCAAGCGGTTCTCAGAAATGGCAGTTACGTGGTTGGGCGTACAACACGATGTTGTCACAA 2560

sheep-CVUAS_3361 equi WGS TGAGGTTTTCCGCGACTTGTCCAAGCGGTTCTCAGAAATGGCAGTTACGTGGTTGGGCGTACAACACGATGTTGTCACAA 2560

alpaca-CVUAS_32656 ovis WGS GCCCAAGTCATCATGATTCTCCCGATGAGATGACCATTGCCAACGGCATAGTTCCAGATATCAACAAAGTTGGTTATGTA 2640

alpaca-CVUAS_32689 ovis WGS GCCCAAGTCATCATGATTCTCCCGATGAGATGACCATTGCCAACGGCATAGTTCCAGATATCAACAAAGTTGGTTATGTA 2640

alpaca-CVUAS_32746 ovis WGS GCCCAAGTCATCATGATTCTCCCGATGAGATGACCATTGCCAACGGCATAGTTCCAGATATCAACAAAGTTGGTTATGTA 2640

alpaca-CVUAS_32834.2 ovis WGS GCCCAAGTCATCATGATTCTCCCGATGAGATGACCATTGCCAACGGCATAGTTCCAGATATCAACAAAGTTGGTTATGTA 2640

alpaca-CVUAS_32842 ovis Sanger GCCCAAGTCATCATGATTCTCCCGATGAGATGACCATTGCCAACGGCATAGTTCCAGATATCAACAAAGTTGGTTATGTA 132

alpaca-CVUAS_32842 ovis WGS GCCCAAGTCATCATGATTCTCCCGATGAGATGACCATTGCCAACGGCATAGTTCCAGATATCAACAAAGTTGGTTATGTA 2640

alpaca-CVUAS_33314 ovis WGS GCCCAAGTCATCATGATTCTCCCGATGAGATGACCATTGCCAACGGCATAGTTCCAGATATCAACAAAGTTGGTTATGTA 2640

camel-KL_293 ovis WGS GCCCAAGTCATCATGATTCTCCCGATGAGATGACCATTGCCAACGGCATAGTTCCAGATATCAACAAAGTTGGTTATGTA 2640

camel-KL_302 ovis WGS GCCCAAGTCATCATGATTCTCCCGATGAGATGACCATTGCCAACGGCATAGTTCCAGATATCAACAAAGTTGGTTATGTA 2640

camel-KL_359 ovis WGS GCCCAAGTCATCATGATTCTCCCGATGAGATGACCATTGCCAACGGCATAGTTCCAGATATCAACAAAGTTGGTTATGTA 2640

dromedary-CVUAS_5583.2 ovis WGS GCCCAAGTCATCATGATTCTCCCGATGAGATGACCATTGCCAACGGCATAGTTCCAGATATCAACAAAGTTGGTTATGTA 2640

horse-ATCC43924 equi Sanger GCCCAAGTCATCATGATTCTCCCGATGAGATGACCATTGCCAACGGCATAGTTCCAGATATCAACAAAGTTGGTTATGTA 132

horse-NC_017945 equi WGS GCCCAAGTCATCATGATTCTCCCGATGAGATGACCATTGCCAACGGCATAGTTCCAGATATCAACAAAGTTGGTTATGTA 2640

llama-191000189 ovis Sanger GCCCAAGTCATCATGATTCTCCCGATGAGATGACCATTGCCAACGGCATAGTTCCAGATATCAACAAAGTTGGTTATGTA 132

llama-191000189 ovis WGS GCCCAAGTCATCATGATTCTCCCGATGAGATGACCATTGCCAACGGCATAGTTCCAGATATCAACAAAGTTGGTTATGTA 2640

llama-CVUAS_4258.2 ovis Sanger GCCCAAGTCATCATGATTCTCCCGATGAGATGACCATTGCCAACGGCATAGTTCCAGATATCAACAAAGTTGGTTATGTA 132

llama-CVUAS_4258.2 ovis WGS GCCCAAGTCATCATGATTCTCCCGATGAGATGACCATTGCCAACGGCATAGTTCCAGATATCAACAAAGTTGGTTATGTA 2640

sheep-CVUAS_3337 equi WGS GCCCAAGTCATCATGATTCTCCCGATGAGATGACCATTGCCAACGGCATAGTTCCAGATATCAACAAAGTTGGTTATGTA 2640

sheep-CVUAS_3338 equi WGS GCCCAAGTCATCATGATTCTCCCGATGAGATGACCATTGCCAACGGCATAGTTCCAGATATCAACAAAGTTGGTTATGTA 2640

sheep-CVUAS_3357 equi Sanger GCCCAAGTCATCATGATTCTCCCGATGAGATGACCATTGCCAACGGCATAGTTCCAGATATCAACAAAGTTGGTTATGTA 132

sheep-CVUAS_3357 equi WGS GCCCAAGTCATCATGATTCTCCCGATGAGATGACCATTGCCAACGGCATAGTTCCAGATATCAACAAAGTTGGTTATGTA 2640

sheep-CVUAS_3361 equi WGS GCCCAAGTCATCATGATTCTCCCGATGAGATGACCATTGCCAACGGCATAGTTCCAGATATCAACAAAGTTGGTTATGTA 2640

alpaca-CVUAS_32656 ovis WGS CCTGGTTTAACCATGCCCAAGATGGCTGTGGTGGAACGCGACTATTCCAAGATCTACGAA-AATGGATGCATTTGGGCCC 2719

alpaca-CVUAS_32689 ovis WGS CCTGGTTTAACCATGCCCAAGATGGCTGTGGTGGAACGCGACTATTCCAAGATCTACGAA-AATGGATGCATTTGGGCCC 2719

alpaca-CVUAS_32746 ovis WGS CCTGGTTTAACCATGCCCAAGATGGCTGTGGTGGAACGCGACTATTCCAAGATCTACGAA-AATGGATGCATTTGGGCCC 2719

alpaca-CVUAS_32834.2 ovis WGS CCTGGTTTAACCATGCCCAAGATGGCTGTGGTGGAACGCGACTATTCCAAGATCTACGAA-AATGGATGCATTTGGGCCC 2719

alpaca-CVUAS_32842 ovis Sanger CCTGGTTTAACCATGCCCAAGATGGCTGTGGTGGAACGCGACTATTCCAAGATCTACGAA-AATGGATGCATTTGGGCCC 211

alpaca-CVUAS_32842 ovis WGS CCTGGTTTAACCATGCCCAAGATGGCTGTGGTGGAACGCGACTATTCCAAGATCTACGAA-AATGGATGCATTTGGGCCC 2719

alpaca-CVUAS_33314 ovis WGS CCTGGTTTAACCATGCCCAAGATGGCTGTGGTGGAACGCGACTATTCCAAGATCTACGAA-AATGGATGCATTTGGGCCC 2719

camel-KL_293 ovis WGS CCTGGTTTAACCATGCCCAAGATGGCTGTGGTGGAACGCGACTATTCCAAGATCTACGAA-AATGGATGCATTTGGGCCC 2719

camel-KL_302 ovis WGS CCTGGTTTAACCATGCCCAAGATGGCTGTGGTGGAACGCGACTATTCCAAGATCTACGAA-AATGGATGCATTTGGGCCC 2719

camel-KL_359 ovis WGS CCTGGTTTAACCATGCCCAAGATGGCTGTGGTGGAACGCGACTATTCCAAGATCTACGAA-AATGGATGCATTTGGGCCC 2719

dromedary-CVUAS_5583.2 ovis WGS CCTGGTTTAACCATGCCCAAGATGGCTGTGGTGGAACGCGACTATTCCAAGATCTACGAA-AATGGATGCATTTGGGCCC 2719

horse-ATCC43924 equi Sanger CCTGGTTTAACCATGCCCAAGATGGCTGTGGTGGAACGCGACTATTCCAAGATCTACGAAAAATGGATGCATTTGGGCCC 212

horse-NC_017945 equi WGS CCTGGTTTAACCATGCCCAAGATGGCTGTGGTGGAACGCGACTATTCCAAGATCTACGAAAAATGGATGCATTTGGGCCC 2720

llama-191000189 ovis Sanger CCTGGTTTAACCATGCCCAAGATGGCTGTGGTGGAACGCGACTATTCCAAGATCTACGAAAAATGGATGCATTTGGGCCC 212

llama-191000189 ovis WGS CCTGGTTTAACCATGCCCAAGATGGCTGTGGTGGAACGCGACTATTCCAAGATCTACGAAAAATGGATGCATTTGGGCCC 2720

llama-CVUAS_4258.2 ovis Sanger CCTGGTTTAACCATGCCCAAGATGGCTGTGGTGGAACGCGACTATTCCAAGATCTACGAA-AATGGATGCATTTGGGCCC 211

llama-CVUAS_4258.2 ovis WGS CCTGGTTTAACCATGCCCAAGATGGCTGTGGTGGAACGCGACTATTCCAAGATCTACGAA-AATGGATGCATTTGGGCCC 2719

sheep-CVUAS_3337 equi WGS CCTGGTTTAACCATGCCCAAGATGGCTGTGGTGGAACGCGACTATTCCAAGATCTACGAAAAATGGATGCATTTGGGCCC 2720

sheep-CVUAS_3338 equi WGS CCTGGTTTAACCATGCCCAAGATGGCTGTGGTGGAACGCGACTATTCCAAGATCTACGAAAAATGGATGCATTTGGGCCC 2720

sheep-CVUAS_3357 equi Sanger CCTGGTTTAACCATGCCCAAGATGGCTGTGGTGGAACGCGACTATTCCAAGATCTACGAAAAATGGATGCATTTGGGCCC 212

sheep-CVUAS_3357 equi WGS CCTGGTTTAACCATGCCCAAGATGGCTGTGGTGGAACGCGACTATTCCAAGATCTACGAAAAATGGATGCATTTGGGCCC 2720

sheep-CVUAS_3361 equi WGS CCTGGTTTAACCATGCCCAAGATGGCTGTGGTGGAACGCGACTATTCCAAGATCTACGAAAAATGGATGCATTTGGGCCC 2720

Consensus ATTGCCGGCGTCGGCAGGCACTGCGGTACATGGCACAAAGTTCGATGTATCCAAACAGGTCGAAGAAATTGCTGCAATAA 2800

alpaca-CVUAS_32656 ovis WGS ATTGCCGGCGTCGGCAGGCACTGCGGTACATGGCACAAAGTTCGATGTATCCAAACAGGTCGAAGAAATTGCTGCAATAA 2799

alpaca-CVUAS_32689 ovis WGS ATTGCCGGCGTCGGCAGGCACTGCGGTACATGGCACAAAGTTCGATGTATCCAAACAGGTCGAAGAAATTGCTGCAATAA 2799

alpaca-CVUAS_32746 ovis WGS ATTGCCGGCGTCGGCAGGCACTGCGGTACATGGCACAAAGTTCGATGTATCCAAACAGGTCGAAGAAATTGCTGCAATAA 2799

alpaca-CVUAS_32834.2 ovis WGS ATTGCCGGCGTCGGCAGGCACTGCGGTACATGGCACAAAGTTCGATGTATCCAAACAGGTCGAAGAAATTGCTGCAATAA 2799

alpaca-CVUAS_32842 ovis Sanger ATTGCCGGCGTCGGCAGGCACTGCGGTACATGGCACAAAGTTCGATGTATCCAAACAGGTCGAAGAAATTGCTGCAATAA 291

alpaca-CVUAS_32842 ovis WGS ATTGCCGGCGTCGGCAGGCACTGCGGTACATGGCACAAAGTTCGATGTATCCAAACAGGTCGAAGAAATTGCTGCAATAA 2799

alpaca-CVUAS_33314 ovis WGS ATTGCCGGCGTCGGCAGGCACTGCGGTACATGGCACAAAGTTCGATGTATCCAAACAGGTCGAAGAAATTGCTGCAATAA 2799

camel-KL_293 ovis WGS ATTGCCGGCGTCGGCAGGCACTGCGGTACATGGCACAAAGTTCGATGTATCCAAACAGGTCGAAGAAATTGCTGCAATAA 2799

camel-KL_302 ovis WGS ATTGCCGGCGTCGGCAGGCACTGCGGTACATGGCACAAAGTTCGATGTATCCAAACAGGTCGAAGAAATTGCTGCAATAA 2799

camel-KL_359 ovis WGS ATTGCCGGCGTCGGCAGGCACTGCGGTACATGGCACAAAGTTCGATGTATCCAAACAGGTCGAAGAAATTGCTGCAATAA 2799

dromedary-CVUAS_5583.2 ovis WGS ATTGCCGGCGTCGGCAGGCACTGCGGTACATGGCACAAAGTTCGATGTATCCAAACAGGTCGAAGAAATTGCTGCAATAA 2799

horse-ATCC43924 equi Sanger CTTGCCGGCGTCGGCAGGCACTGCGGTACATGGCACAAAGTTCGATGTATCCAAACAGGTCGAAGAAATTGCTGCAATAA 292

horse-NC_017945 equi WGS CTTGCCGGCGTCGGCAGGCACTGCGGTACATGGCACAAAGTTCGATGTATCCAAACAGGTCGAAGAAATTGCTGCAATAA 2800

llama-191000189 ovis Sanger ATTGCCGGCGTCGGCAGGCACTGCGGTACATGGCACAAAGTTCGATGTATCCAAACAGGTCGAAGAAATTGCTGCAATAA 292

llama-191000189 ovis WGS ATTGCCGGCGTCGGCAGGCACTGCGGTACATGGCACAAAGTTCGATGTATCCAAACAGGTCGAAGAAATTGCTGCAATAA 2800

llama-CVUAS_4258.2 ovis Sanger ATTGCCGGCGTCGGCAGGCACTGCGGTACATGGCACAAAGTTCGATGTATCCAAACAGGTCGAAGAAATTGCTGCAATAA 291

llama-CVUAS_4258.2 ovis WGS ATTGCCGGCGTCGGCAGGCACTGCGGTACATGGCACAAAGTTCGATGTATCCAAACAGGTCGAAGAAATTGCTGCAATAA 2799

sheep-CVUAS_3337 equi WGS ATTGCCGGCGTCGGCAGGCACTGCGGTACATGGCACAAAGTTCGATGTATCCAAACAGGTCGAAGAAATTGCTGCAATAA 2800

sheep-CVUAS_3338 equi WGS ATTGCCGGCGTCGGCAGGCACTGCGGTACATGGCACAAAGTTCGATGTATCCAAACAGGTCGAAGAAATTGCTGCAATAA 2800

sheep-CVUAS_3357 equi Sanger ATTGCCGGCGTCGGCAGGCACTGCGGTACATGGCACAAAGTTCGATGTATCCAAACAGGTCGAAGAAATTGCTGCAATAA 292

sheep-CVUAS_3357 equi WGS ATTGCCGGCGTCGGCAGGCACTGCGGTACATGGCACAAAGTTCGATGTATCCAAACAGGTCGAAGAAATTGCTGCAATAA 2800

sheep-CVUAS_3361 equi WGS ATTGCCGGCGTCGGCAGGCACTGCGGTACATGGCACAAAGTTCGATGTATCCAAACAGGTCGAAGAAATTGCTGCAATAA 2800

alpaca-CVUAS_32656 ovis WGS ACGGGGTCAACGAAATCTCTTTTGGTCCCCGCCCAAGGTTAGATACCGCAATCAAGGTTATTAACGCGATCTTGCATATG 2879

alpaca-CVUAS_32689 ovis WGS ACGGGGTCAACGAAATCTCTTTTGGTCCCCGCCCAAGGTTAGATACCGCAATCAAGGTTATTAACGCGATCTTGCATATG 2879

alpaca-CVUAS_32746 ovis WGS ACGGGGTCAACGAAATCTCTTTTGGTCCCCGCCCAAGGTTAGATACCGCAATCAAGGTTATTAACGCGATCTTGCATATG 2879

alpaca-CVUAS_32834.2 ovis WGS ACGGGGTCAACGAAATCTCTTTTGGTCCCCGCCCAAGGTTAGATACCGCAATCAAGGTTATTAACGCGATCTTGCATATG 2879

alpaca-CVUAS_32842 ovis Sanger ACGGGGTCAACGAAATCTCTTTTGGTCCCCGCCCAAGGTTAGATACCGCAATCAAGGTTATTAACGCGATCTTGCATATG 371

alpaca-CVUAS_32842 ovis WGS ACGGGGTCAACGAAATCTCTTTTGGTCCCCGCCCAAGGTTAGATACCGCAATCAAGGTTATTAACGCGATCTTGCATATG 2879

alpaca-CVUAS_33314 ovis WGS ACGGGGTCAACGAAATCTCTTTTGGTCCCCGCCCAAGGTTAGATACCGCAATCAAGGTTATTAACGCGATCTTGCATATG 2879

camel-KL_293 ovis WGS ACGGGGTCAACGAAATCTCTTTTGGTCCCCGCCCAAGGTTAGATACCGCAATCAAGGTTATTAACGCGATCTTGCATATG 2879

camel-KL_302 ovis WGS ACGGGGTCAACGAAATCTCTTTTGGTCCCCGCCCAAGGTTAGATACCGCAATCAAGGTTATTAACGCGATCTTGCATATG 2879

camel-KL_359 ovis WGS ACGGGGTCAACGAAATCTCTTTTGGTCCCCGCCCAAGGTTAGATACCGCAATCAAGGTTATTAACGCGATCTTGCATATG 2879

dromedary-CVUAS_5583.2 ovis WGS ACGGGGTCAACGAAATCTCTTTTGGTCCCCGCCCAAGGTTAGATACCGCAATCAAGGTTATTAACGCGATCTTGCATATG 2879

horse-ATCC43924 equi Sanger ACGGGGTCAACGAAACCTCTTTTGGTCCCCGCCCAAGACTAGATACCGCAATCAAGGTTATTAACGCGATCTTGCATATG 372

horse-NC_017945 equi WGS ACGGGGTCAACGAAACCTCTTTTGGTCCCCGCCCAAGACTAGATACCGCAATCAAGGTTATTAACGCGATCTTGCATATG 2880

llama-191000189 ovis Sanger ACGGGGTCAACGAAATCTCTTTTGGTCCCCGCCCAAGGTTAGATACCGCAATCAAGGTTATTAACGCGATCTTGCATATG 372

llama-191000189 ovis WGS ACGGGGTCAACGAAATCTCTTTTGGTCCCCGCCCAAGGTTAGATACCGCAATCAAGGTTATTAACGCGATCTTGCATATG 2880

llama-CVUAS_4258.2 ovis Sanger ACGGGGTCAACGAAATCTCTTTTGGTCCCCGCCCAAGGTTAGATACCGCAATCAAGGTTATTAACGCGATCTTGCATATG 371

llama-CVUAS_4258.2 ovis WGS ACGGGGTCAACGAAATCTCTTTTGGTCCCCGCCCAAGGTTAGATACCGCAATCAAGGTTATTAACGCGATCTTGCATATG 2879

sheep-CVUAS_3337 equi WGS ACGGGGTCAACGAAATCTCTTTTGGTCCCCGCCCAAGGTTAGATACCGCAATCAAGGTTATTAACGCGATCTTGCATATG 2880

sheep-CVUAS_3338 equi WGS ACGGGGTCAACGAAATCTCTTTTGGTCCCCGCCCAAGGTTAGATACCGCAATCAAGGTTATTAACGCGATCTTGCATATG 2880

sheep-CVUAS_3357 equi Sanger ACGGGGTCAACGAAATCTCTTTTGGTCCCCGCCCAAGGTTAGATACCGCAATCAAGGTTATTAACGCGATCTTGCATATG 372

sheep-CVUAS_3357 equi WGS ACGGGGTCAACGAAATCTCTTTTGGTCCCCGCCCAAGGTTAGATACCGCAATCAAGGTTATTAACGCGATCTTGCATATG 2880

sheep-CVUAS_3361 equi WGS ACGGGGTCAACGAAATCTCTTTTGGTCCCCGCCCAAGGTTAGATACCGCAATCAAGGTTATTAACGCGATCTTGCATATG 2880

alpaca-CVUAS_32656 ovis WGS TCGGGAGTATCTAACGGTGAAGTTGCCGCTGAAGGCTTTAAGTTCTTGTCCAAACGCACTGGTAAAGATCTCACGGTGCT 2959

alpaca-CVUAS_32689 ovis WGS TCGGGAGTATCTAACGGTGAAGTTGCCGCTGAAGGCTTTAAGTTCTTGTCCAAACGCACTGGTAAAGATCTCACGGTGCT 2959

alpaca-CVUAS_32746 ovis WGS TCGGGAGTATCTAACGGTGAAGTTGCCGCTGAAGGCTTTAAGTTCTTGTCCAAACGCACTGGTAAAGATCTCACGGTGCT 2959

alpaca-CVUAS_32834.2 ovis WGS TCGGGAGTATCTAACGGTGAAGTTGCCGCTGAAGGCTTTAAGTTCTTGTCCAAACGCACTGGTAAAGATCTCACGGTGCT 2959

alpaca-CVUAS_32842 ovis Sanger TCGGG--------------------------------------------------------------------------- 451

alpaca-CVUAS_32842 ovis WGS TCGGGAGTATCTAACGGTGAAGTTGCCGCTGAAGGCTTTAAGTTCTTGTCCAAACGCACTGGTAAAGATCTCACGGTGCT 2959

alpaca-CVUAS_33314 ovis WGS TCGGGAGTATCTAACGGTGAAGTTGCCGCTGAAGGCTTTAAGTTCTTGTCCAAACGCACTGGTAAAGATCTCACGGTGCT 2959

camel-KL_293 ovis WGS TCGGGAGTATCTAACGGTGAAGTTGCCGCTGAAGGCTTTAAGTTCTTGTCCAAACGCACTGGTAAAGATCTCACGGTGCT 2959

camel-KL_302 ovis WGS TCGGGAGTATCTAACGGTGAAGTTGCCGCTGAAGGCTTTAAGTTCTTGTCCAAACGCACTGGTAAAGATCTCACGGTGCT 2959

camel-KL_359 ovis WGS TCGGGAGTATCTAACGGTGAAGTTGCCGCTGAAGGCTTTAAGTTCTTGTCCAAACGCACTGGTAAAGATCTCACGGTGCT 2959

dromedary-CVUAS_5583.2 ovis WGS TCGGGAGTATCTAACGGTGAAGTTGCCGCTGAAGGCTTTAAGTTCTTGTCCAAACGCACTGGTAAAGATCTCACGGTGCT 2959

horse-ATCC43924 equi Sanger TCGGG--------------------------------------------------------------------------- 452

horse-NC_017945 equi WGS TCGGGAGTATCTAACGGTGAAGTTGCCGCTGAAGGCTTTAAGTTCTTATCCAAACGCACTGGTAAAGATCTCACGGTGCT 2960

llama-191000189 ovis Sanger TCGGG--------------------------------------------------------------------------- 452

llama-191000189 ovis WGS TCGGGAGTATCTAACGGTGAAGTTGCCGCTGAAGGCTTTAAGTTCTTGTCCAAACGCACTGGTAAAGATCTCACGGTGCT 2960

llama-CVUAS_4258.2 ovis Sanger TCGGG--------------------------------------------------------------------------- 451

llama-CVUAS_4258.2 ovis WGS TCGGGAGTATCTAACGGTGAAGTTGCCGCTGAAGGCTTTAAGTTCTTGTCCAAACGCACTGGTAAAGATCTCACGGTGCT 2959

sheep-CVUAS_3337 equi WGS TCGGGAGTATCTAACGGTGAAGTTGCCGCTGAAGGCTTTAAGTTCTTGTCCAAACGCACTGGTAAAGATCTCACGGTGCT 2960

sheep-CVUAS_3338 equi WGS TCGGGAGTATCTAACGGTGAAGTTGCCGCTGAAGGCTTTAAGTTCTTGTCCAAACGCACTGGTAAAGATCTCACGGTGCT 2960

sheep-CVUAS_3357 equi Sanger TCGGG--------------------------------------------------------------------------- 452

sheep-CVUAS_3357 equi WGS TCGGGAGTATCTAACGGTGAAGTTGCCGCTGAAGGCTTTAAGTTCTTGTCCAAACGCACTGGTAAAGATCTCACGGTGCT 2960

sheep-CVUAS_3361 equi WGS TCGGGAGTATCTAACGGTGAAGTTGCCGCTGAAGGCTTTAAGTTCTTGTCCAAACGCACTGGTAAAGATCTCACGGTGCT 2960
