# Supplementary material for: Corynebacterium pseudotuberculosis: Whole genome sequencing reveals unforeseen and relevant genetic diversity in this pathogen
Source: PLoS One. 2024 Aug 26;19(8):e0309282. doi: 10.1371/journal.pone.0309282 (PMC11346948; doi:10.1371/journal.pone.0309282)
Supplement: S1 File — SNP detected by alignment of the different isolates included in this study are labeled. (DOCX) [file pone.0309282.s016.docx]

ATGAAAATGAAGAAACTCGGATTGGCAGTTGCCACCCTGACCGTTGCCGCGACTCTGTCT

GGTTGTTTTACCGACTCAGGTTCTGACTCCACCGACGGACAGACCTTGCGCGTTGCGCTG

CAGTTCAAGCCCGTCGCTGATTTCTCCCCGTTCTCCGACGACTCCGTGCTTAACCTGCGC

ATGGGGGTCGCGGAGACCCTGGTAACCCTTGATGAGGATGCAAAGCTTAAGCCAGTACTC

GCTGAGAAGTGGGAGATGAAGGATGACCGCACTGCTGTGCTTAGTCTGCGCCAGGGCGTG

ACATTCCACGATGGAAGCAAACTCGACGCAAAGGCCGTGA-AAATGCTCTCGACCACGCT

CTTTCCGCAGCTACCCGACCAAAGGGACTCGGTAAGGCAGACCTCAAGGTTGAGGCCACC

GGCGAGCATGAGGTGACCGTCACGTCGCCCAAGGCTGACCCCATTTTGGTTCAGCGCTTC

TCTGACCCCGGCACCGCCATCCTTGCTCAAGCCGCATACAAAGGGGAGAATTCCGACCCG

TTTGCCCACGGGACAGGGCCCTTCAAGCTGGTCAAGAAAGAGACCGACGGTTCCGTCACC

GCTGAGGCCTTTGGCGACTATTGGAACGGCAAACCCAAGACCTCCGCTCTGAAAGTCTCC

TTCATTGAAGACGGCGCAGCCCGCGCCAATGCTTTCCGCGCTGGTGACCTGGACGTGGTT

AAGGGAGTCCCCGTGGTCTCGTTGCCAGAGCTTTCCGACGCCCACATCACCGACGTCCAC

CTCCCACGCGCCGTGCTGCTCCACCTCAACGCTGAAAAGGGCGTGTTTGCAGACGCCCAC

CTGCGTGCTGCTGTCGCGGGTGCCATCAAGACCGAGCCCATCGTGGAGAAAATCTACGAG

GGTAAAGCCAACAAGACGCAAAGCTCTCTCTTCAACCAGGACACCGAGTGGGCTGCAGCT

CAGAAGGCTAAGTCGCTTGCAACGGAGGCTCCTAAGGCCGATGCGACTCTCGGGGCGGGC

AAGACTGTTCGCCTAGCTACCTGGGACTCTCGCGCGGAGCTCCCCGAAACCGCG-AATCT

CATCGCCGACCAGCTTCGTGCTTTGGGCTTTAACGTGGAAATCACGGTCGCCGATTACGC

GTCCTTGGAAAAACAGCTTCTCGACGGCTCCTTTGATGCCATCATCGGATCCCGCAACTA

CATGTTTGGCGCCGGCGATCCTCTTGCATTCCTAGAGACCGACTTCTCTTGCGAGGGAGC

TTATAACCTATCGCGCCTCTGTGATCCGAAGATCGACAAAGAAATTGCCGACGCTAAGGG

CCAGAAAGACCTCAACACCCGTCTCCAGAACGCCGCATCCATCGGCGCAGACATCGTGTC

CACCGGAGCCGTCGTCCCACTGGCCCACGAGCAATTGCTGATCGCTTCTAAAAACGTAGA

GGGCCTGTCTACCGATCCCATGGAGCGTTCCCTGATCACAGAAAAGACGGAACTTTCCGC

TAAGAAGTAA
